# Correlates of prenatal and postnatal mother-to-infant bonding quality: A systematic review

**DOI:** 10.1371/journal.pone.0222998

**Published:** 2019-09-24

**Authors:** Elke Tichelman, Myrte Westerneng, Anke B. Witteveen, Anneloes L. van Baar, Henriëtte E. van der Horst, Ank de Jonge, Marjolein Y. Berger, François G. Schellevis, Huibert Burger, Lilian L. Peters

**Affiliations:** 1 Amsterdam UMC, Vrije Universiteit Amsterdam, Midwifery Science, AVAG, Amsterdam Public Health Research Institute, Amsterdam, the Netherlands; 2 University of Groningen, University Medical Centre Groningen, Department of General Practice & Elderly Care Medicine, Groningen, the Netherlands; 3 Child and Adolescent Studies, Utrecht University, Utrecht, the Netherlands; 4 Amsterdam UMC, Vrije Universiteit Amsterdam, Department of General Practice & Elderly Care Medicine, Amsterdam Public Health Research Institute, Amsterdam, the Netherlands; 5 NIVEL, Netherlands Institute for Health Services Research, Utrecht, the Netherlands; London School of Hygiene and Tropical Medicine, UNITED KINGDOM

## Abstract

**Background:**

Mother-to-infant bonding is defined as the emotional tie experienced by a mother towards her child, which is considered to be important for the socio-emotional development of the child. Numerous studies on the correlates of both prenatal and postnatal mother-to-infant bonding quality have been published over the last decades. An up-to-date systematic review of these correlates is lacking, however.

**Objective:**

To systematically review correlates of prenatal and postnatal mother-to-infant bonding quality in the general population, in order to enable targeted interventions.

**Methods:**

MEDLINE, Embase, CINAHL, and PsychINFO were searched through May 2018. Reference checks were performed. Case-control, cross-sectional or longitudinal cohort studies written in English, German, Swedish, Spanish, Norwegian, French or Dutch defining mother-to-infant bonding quality as stipulated in the protocol (PROSPERO CRD42016040183) were included. Two investigators independently reviewed abstracts, full-text articles and extracted data. Methodological quality was assessed using the National Institute of Health Quality Assessment Tool for Observational Cohort and Cross-sectional studies and was rated accordingly as poor, fair or good. Clinical and methodological heterogeneity were examined.

**Main results:**

131 studies were included. Quality was fair for 20 studies, and poor for 111 studies. Among 123 correlates identified, 3 were consistently associated with mother-to-infant bonding quality: 1) duration of gestation at assessment was positively associated with prenatal bonding quality, 2) depressive symptoms were negatively associated with postnatal mother-to-infant bonding quality, and 3) mother-to-infant bonding quality earlier in pregnancy or postpartum was positively associated with mother-to-infant bonding quality later in time.

**Conclusion:**

Our review suggests that professionals involved in maternal health care should consider monitoring mother-to-infant bonding already during pregnancy. Future research should evaluate whether interventions aimed at depressive symptoms help to promote mother-to-infant bonding quality. More high-quality research on correlates for which inconsistent results were found is needed.

## Introduction

Mother-to-infant bonding is defined as the emotions and feelings experienced by a mother towards her child [1,2]. Mother-to-infant bonding should not be confused with attachment, which refers to the connectedness between an infant and a caregiver characterized by the child using its caregiver as a secure base for exploration [3]. Mother-to-infant bonding, unlike attachment, is unidirectional (from mother to child) and starts to develop already during pregnancy, after which it further develops until early childhood [1,4,5].

Adequate mother-to-infant bonding is considered to be important for a positive socio-emotional development of the child [6]. A poorer mother-to-infant bond is associated with less sensitive and poorer maternal parenting styles and skills [7,8], possibly due to lowered neural sensitivity to infant‐related stimuli (e.g. facial cues) in these mothers [9–11]. Less sensitive, and poorer maternal parenting, in turn, has been linked to insecure attachment [12], depression [13–16], and anxiety in children [13]. Furthermore, it has been found that women with a poorer mother-to-infant bond show less interest in their child’s health, and engage more frequently in negative health behaviors during pregnancy [17]. These behaviors are associated with adverse birth outcomes, and adverse long-term cognitive and socio-emotional development of the child [18].

To prevent these unfavourable outcomes, strategies to strengthen mother-to-infant bonding quality are required. To develop such strategies, a systematic review of correlates of mother-to-infant bonding quality is an essential first step. The four reviews that have been performed thus far, of which one met the criteria of a systematic review, focused on prenatal bonding only [19–22]. Also, these reviews are at least nine years old. Numerous studies on mother-to-infant bonding have been published since, requiring an update of the current state of knowledge.

The aim of our systematic review, therefore, is to provide an up-to-date overview of correlates of both prenatal and postnatal mother-to-infant bonding quality in the general population.

## Methods

Our librarian-assisted search (S1 File) was performed in MEDLINE, Embase, CINAHL and PsychINFO for studies published prior to May 7th 2018 using search terms referring to (1) attachment, bonding, parent-child relations; (2) pregnancy, mother, infant, newborn; (3) mother-to-infant bonding instruments which, in accordance with the definition of mother-to-infant bonding, focus on the emotions and feelings experienced by a mother towards her child (S1 Table). The list of mother-to-infant bonding instruments was composed by scanning the first 2000 papers that resulted from combining search terms 1 and 2 described above. Mother-to-infant bonding instruments that were initially not included in our list, but came to our attention later on through reference checks or recently papers published, were added to the search. Subscales of mother-to-infant bonding instruments (e.g. rejection and anger) were not considered representative of the overall construct of mother-to-infant bonding and therefore excluded. We included (1) full texts, (2) written in English, German, Swedish, Spanish, Norwegian, French or Dutch; (3) case-control, cross-sectional or (retro- or prospective) longitudinal cohort studies / study designs as stipulated in the protocol (PROSPERO CRD42016040183) (S2 File). Duplicate and qualitative studies were excluded. Furthermore, we excluded experimental studies because an overview of interventions to optimize mother-to-infant bonding has been described elsewhere [23]. Finally, we limited our systematic review to the general population by excluding studies focusing on specific subgroups only (e.g. mothers with teenage pregnancies) as we expected different factors to be associated with mother-to-infant bonding in these subgroups. After screening titles and abstracts, eligibility was based on full-text versions. New articles were identified through reference checks. Characteristics of the studies (S2 Table) and results on the associations between correlates and mother-to-infant bonding quality in terms of correlation coefficients, regression coefficients, odds ratios and inferential statistics were extracted. Results were considered to be significant at a p-value < 0.05. We considered correlations between 0–0.29 as weak, 0.3–0.59 as moderate and 0.6–1 as strong [24]. Methodological quality was assessed using the National Institute of Health Quality Assessment Tool for Observational Cohort and Cross-sectional studies and rated as poor, fair or good (S3 File) [25]. Screening of titles and abstracts, reviewing and data extraction was done independently by ET and MW. We calculated the percentage of inter-observer agreement and Cohen’s kappa for titles and abstracts screening [26]. A.B.W. resolved discrepancies in reviewing that were not resolved through discussion. Clinical and methodological heterogeneity of the studies were assessed as stipulated in the protocol. The PRISMA statement was followed for reporting [27].

## Results

**Fig 1:**
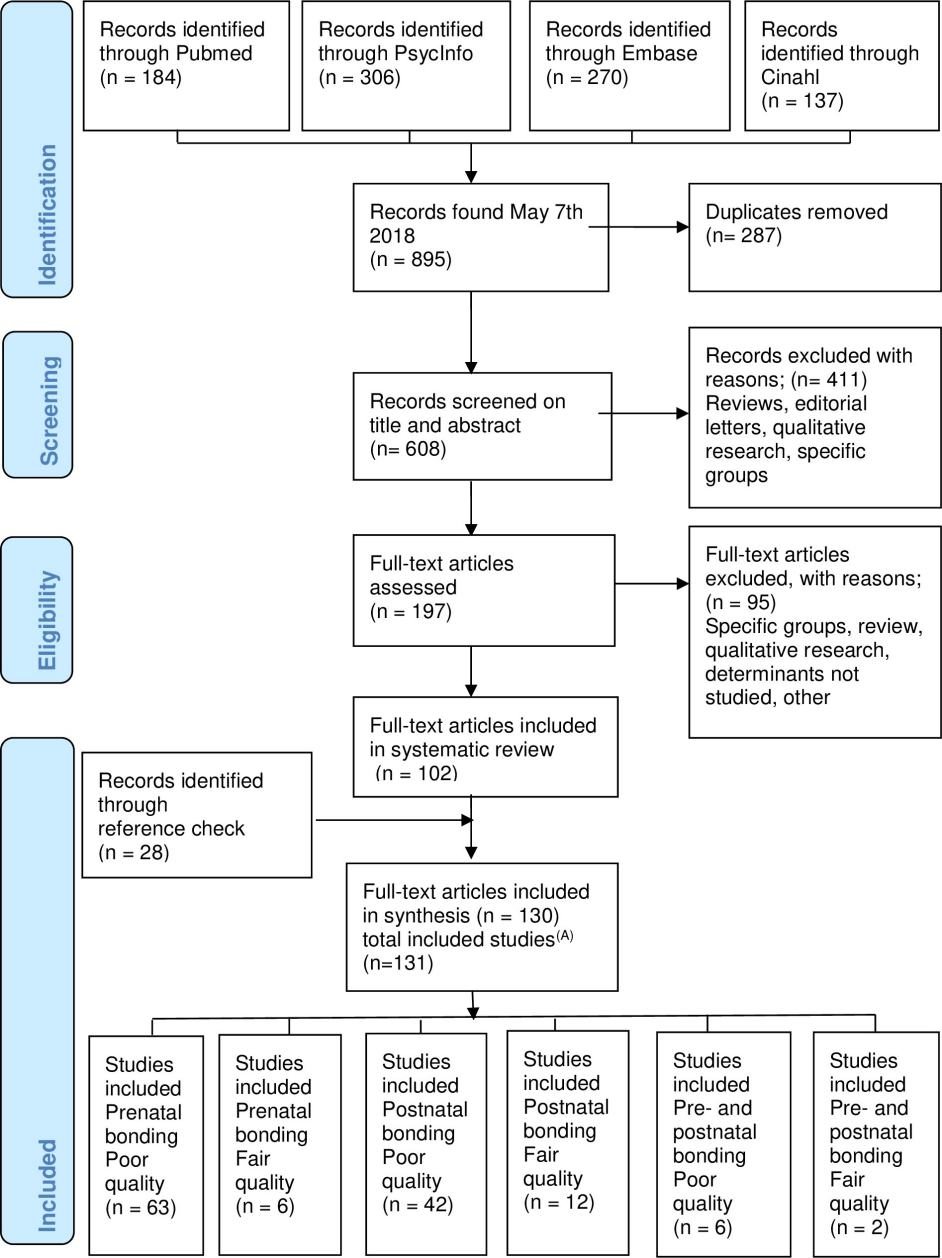
Flowchart of the systematic review. One included article described two different included studies.

The search identified 895 titles, of which 197 were fully reviewed. Agreement for the screening of titles and abstracts was 92% (kappa = 0.81) [26]. In total 130 articles met our inclusion criteria, of which 28 were identified through reference checks. One article described two different studies, resulting in 131 included studies published in the years 1981–2018 (Fig 1). All studies had a cross-sectional or prospective cohort design. Quality was fair for 20 studies, and poor for 111 studies. In 37 (28%) of the 131 included studies, multivariable analyses were performed. A detailed overview of the characteristics of the studies can be found in the supplement (S2 Table).

Mother-to-infant bonding was measured by several instruments. For prenatal assessment, the Maternal Fetal Attachment Scale (MFAS, 46 studies) [28] was used most frequently. For postnatal assessment, the Postpartum Bonding Questionnaire (PBQ or PBQ-S, 24 studies) [29] was used most frequently. In 123 studies (94%) mother-to-infant bonding was treated as a continuous variable. Time of measuring mother-to-infant bonding varied from the first trimester of pregnancy to 24 months postpartum.

We identified 123 correlates of mother-to-infant bonding quality and classified these into 5 categories: demographic, reproduction-related, psychosocial, child-related and partner-related. Partner-related correlates referred to correlates that solely concerned the partner (e.g. partner’s educational level, partner’s attachment to the fetus). Correlates that reflected the relationship with the partner as experienced by the mother were categorized as psychosocial. Across studies, correlates were examined in 1 (e.g. spirituality) to 44 studies (depressive symptoms). Because of the great heterogeneity in measures used for mother-to-infant bonding, operationalization of correlates, time of measuring correlates and outcomes, and analyses performed, data were not suitable for meta-analysis and we therefore summarized them narratively [30].

To provide more reliable results, in accordance with other systematic reviews, we decided to only discuss correlates that were examined in at least 10 studies (either prenatal or postnatal) [20,31]. An overview of these correlates, and whether they were found to be associated with mother-to-infant bonding quality, can be found in Table 1. Each study has been assigned a number, which corresponds with the study numbers in S2 Table and S4 File. Results of the studies which found a significant association are summarized in Tables 2 and 3. A summary of the findings on correlates that were examined in less than 10 studies is provided in the supplement (S3 Table). All identified partner-related correlates were examined in less than 10 studies and are therefore only included in the supplement.

**Table 1:** Studies, identified by number, examining associations between correlates examined in at least ten different studies with mother-to-infant bonding quality.

	Prenatal mother-to-infant bonding					Postnatal mother-to-infant bonding		
Correlates	Association YES	Association NO			Inconclusive	Association YES	Association NO	Inconclusive
*Demographic*								
Maternal age	9,36,**39**,69,80,108, 115,116	**3,5**,7,11,20,21,23, 24,27,43,55,67,88, **98**,124			**99**	**59**,94	2,13,18,**30**,33, **60**,71,83,87,91,113	
Educational level	23,36,80,115	3,**5**,24,27,43,53, 55,67,88			69	12,**28**,33,94	2,18,**30**,71,79,87, 91,113	
Occupation/employment	42,53,88	23,24,115,116			36		2,18,33,87,91,113	
Income	36,67	3,23,27,69,80,116					2,83,87	
Marital status	3,**39**	24,67,80,88			69	91	13,33,**59**	
*Reproduction-related*								
Duration of gestation at assessment	3,**5**,7,11,24,**39**,43, 44,69,77,80,93,106, 108,112, 122,124	27,55,70,**98,**115					91	
Number of children	20,36,**39**,44, 55, 76,**103**	3,7,24,27,43,69, 80,88,**98,**108				12,**59,**109	2,13,**30,**33,**60**,87, 91,113	
Number of pregnancies	36,89,114,115	21,27,67,**98**,124					13	
Planned pregnancy	20,23,67,115	3,24,53,55,88,**98**					**41**,87,91	
Desired pregnancy	53,80,88,**98**,118, 131	24,56				2	91	**60**
Mode of birth						14,49,126	2,13,18,33,87,91	**127**
*Psychosocial*								
Depressive symptoms	11,26,32,73,86,88	**5**,25,27,42,44,53, **103**			20,63,69, 116	12,**29,30,**33,38, 49,52,57,**59,**60, 71,84,85,86,87,91, 94,95,102,109, 113,130	89	**28,**35,79,83, **97,111**
Earlier mother-to-infant bonding	**51,97,98**					11,12,**19,**27,**28, 30,**52,**60,**81,91, 95,**97,**109,**111,** 121		**29**
General attachment	77,110,117	20,27,72,78,101, 124,128					**30**	12,104
General anxiety	32	27,116			20,37	12,**30,**35,83,102		**28,60,97**
Social support	20,21,23,53,56,88	11,108			80		33,91	2,18
Quality of partner relationship	**5**,11,22,23,27,72	80,88,116,124			20	2,**41**	**30,**87,91	
*Child-related*								
Gender baby						**30**,87	2,33,**41**,71,79,83, 94,**111**,113	

Studies in **bold** were of fair methodological quality according the Quality Assessment Tool for Observational Cohort and Cross-sectional studies of the National Heart, Lung, and Blood Institute whereas other studies were of poor quality.

The numbers refer to the different studies. More detailed information about the included studies are reported in S2 Table the references can be found in appendix S4.

The alpha level was set at 0.05 to be classified as a significant association.

In some studies, multiple analysis were performed (e.g. at different time points, with different instruments), which sometimes resulted in inconclusive (both significant and non-significant) results.

**Table 2:** Significant results of studies examining associations between demographic and reproduction-related correlates examined in at least 10 different studies with mother-to-infant bonding quality.

		Prenatal mother-to-infant bonding		Postnatal mother-to-infant bonding	
Correlates	Quality	Number of studies with significant results	Direction of associations of significant study results	Number of studies with significant results	Direction of associations of significant study results
**Demographic**					
Age	poor	7 of 20	Negative associations (r = -0·22 to -0·09, β = -0·80 to -0·15)	1 of 10	Negative associations (r = -0·13 to -0·12)
	fair	2 of 4	Negative association (r = -0·22) and positive association (non-adolescent > adolescents (β = 0·21))	1 of 3	Negative association (β = -0·15)
Education	poor	5 of 13	Negative and positive associations (r = -0·14 to 0·12, higher education > median (K-W))	3 of 10	Positive associations (higher education > means (t-tests and ANOVA))
	fair	0 of 1	-	1 of 2	Negative association (r = - 0·25)
Occupation/ employment	poor	4 of 8	Negative and positive associations (r = -0·19 to 0·25, working/studying > housewife (OR = 3·1), housewife > full time > part time worker (ANOVA))	0 of 6	-
Income	poor	2 of 8	Negative and positive association (r = -0·26 and r = 0·10)	0 of 3	
Marital status	poor	2 of 6	Positive associations (r = 0·13, married > median (MWU))	1 of 3	Positive association (married > single (OR = 7·1))
	fair	1 of 1	Positive association (r = 0·14)	0 of 1	-
**Reproduction-related**					
Duration of gestation at assessment	poor	15 of 19	Positive associations (r = 0·14 to 0·36, β = 0·28 to β = 0·47, higher gestational age > means (t-test and ANOVA))	0 of 1	**-**
	fair	2 of 3	Positive associations (r = 0·11, β = 0·37)	**-**	**-**
Number of children	poor	5 of 14	Negative associations (r = -0·29 to -0·18, β = -0·20 to -0·15)	2 of 8	Negative and positive association (1^st^ child < mean (t-test), 1^st^ child > median (MWU))
	fair	2 of 3	Negative associations (r = -0·14, β = -3·97)	1 of 3	Positive association (β = 0·36)
Number of pregnancies	poor	4 of 8	Negative association (r = -0·42, β = - 0·22 to -0·20, primigravida > means (t-test))	0 of 1	
	fair	0 of 1		-	**-**
Planned pregnancy	poor	4 of 9	Positive associations (r = 0·24 to 0·29, planned pregnancy > median (MWU))	0 of 1	**-**
Desired pregnancy	poor	5 of 7	Positive associations (r = 0·16 to 0·46, desired > not desired (OR = 5·1))	1 of 2	Positive association (β = 0·168)
	fair	1 of 1	Positive association (desired > mean (t-test))	1 of 1	Positive association (r = 0·23 to 0·33)
Mode of birth	poor	-	-	3 of 9	Vaginal birth > mean (t-test), induction of labour < median (K-W)
	fair	-	-	1 of 1	Vaginal birth > mean (t-test)

Higher scores in this table reflect higher mother-to-infant bonding quality. For this purpose, some scores were reversed accordingly. The alpha level was set at 0·05 to be classified as a significant association. Abbreviations: r = correlation B = unstandardized regression coefficient β = standardized regression coefficient MWU = Mann Whitney U test K-W = Kruskal Wallis test OR = odds ratio > = higher scores < = lower scores

**Table 3:** Significant results of studies examining associations between psychosocial and child-related correlates examined in at least 10 different studies with mother-to-infant bonding quality.

		Prenatal mother-to-infant bonding		Postnatal mother-to-infant bonding	
Correlates	Quality	Number of studies with significant results	Direction of associations of significant study results	Number of studies with significant results	Direction of associations of significant study results
**Psychosocial**					
Depressive symptoms	poor	10 of 15	Negative and (one) positive association (r = -0·38 to 0·26, β = -0·52 to -0·19, not depressed > means (t-test), more depressed < less depressed (OR = 1·7))	22 of 23	Negative associations (r = -0·61 to -0·14, β = - 0·39 to -0·26, depressed < median (MWU), depressed < means (t-test), depressed > % bonding disorder, lifetime mood disorder < no lifetime mood disorder (OR = 3·32))
	fair	0 of 2	-	6 of 6	Negative associations (r = -0·46 to -0·29, β = -0·57 to -0·25)
Earlier mother-to-infant bonding	poor	0 of 0	-	8 of 8	Positive associations (r = 0·22 to 0·98, higher prenatal bonding > lower prenatal bonding (OR 1·17))
	fair	3 of 3	Positive associations (r = 0·61 to 0·74)	8 of 8	Positive associations (r = 0·17 to 0·61, β = 0·09 to 0·386)
General attachment	poor	3 of 10	Positive associations (r = 0·18 to 0·33) secure > means (ANOVA)	2 of 2	Positive associations, secure attachment (r = 0·18 to 0·32)
	fair	-		0 of 1	-
General anxiety	poor	3 of 5	Negative associations (r = -0·26 to -0·25) anxiety < means (t-test)	4 of 4	Negative associations (r = -2·60 to -0·16, β = -0·227)
	fair	0 of 0	-	4 of 4	Negative and (one) positive associations (r = -0·32 to -0·15, β = 0·35)
Social support	poor	7 of 9	Positive associations (r = 0·13 to 0·51, more support > means (t-test), support > no support (OR = 1·8))	2 of 4	Negative and positive association (r = 0·34, β = -0·200 - β = 0·190)
Quality of partner relationship	poor	6 of 10	Positive associations (r = 0·08 to 0·45, β = 0·21)	1 of 3	Positive association (β = 0·291)
	fair	1 of 1	Positive association (unstandardized regression coefficient)	1 of 2	Positive association (good quality > means (ANOVA))
**Child-related**					
Gender baby	poor	0 of 0	-	1 of 8	Boy > median (MWU)
	fair	0 of 0	-	1 of 3	Boy > girl (β = 0·08)

Higher scores in this table reflect higher mother-to-infant bonding quality. For this purpose, some scores were reversed accordingly. The alpha level was set at 0·05 to be classified as a significant association. Abbreviations: r = correlation B = unstandardized regression coefficient β = standardized regression coefficient MWU = Mann Whitney U test K-W = Kruskal Wallis test OR = odds ratio > = higher scores < = lower scores

### Correlates

#### Demographic

Demographic correlates were found to be associated with pre- and postnatal mother-to-infant bonding quality in only a few studies. Studies showed positive associations with being married (4 of 11 studies), and negative associations with increased maternal age (10 of 37 studies), with the exception of one study, which showed that adolescent mothers (<20 years old) had lower prenatal bonding quality than non-adolescent mothers [32]. For educational level, occupation/employment, and income, a minority of the studies found an association (9 of 26, 4 of 14 and 2 of 11 respectively). About half of these studies showed that a higher educational level, working or studying and a higher income level were associated with higher mother-to-infant bonding quality, while the other half of the associations showed the opposite (e.g. being a housewife or unemployed was associated with higher mother-to-infant bonding quality). For all of the above-mentioned demographic variables, reported correlations were weak.

#### Reproduction-related

Duration of gestation at assessment was positively associated with mother-to-infant bonding quality in the vast majority of studies (17 of 23 studies). Women assessed in a later stage of pregnancy had better mother-to-infant bonding quality than women assessed earlier in pregnancy. Correlations were weak or moderate. Mostly weak positive correlations were found between a desired pregnancy and prenatal mother-to-infant bonding quality (6 of 8 studies). Postnatal bonding was only examined in three studies, which showed mixed results regarding an association with a desired pregnancy. Number of children, number of previous pregnancies and planned pregnancy were associated with mother-to-infant bonding quality in some studies: women expecting a second or third child, women with a higher number of previous pregnancies, or women who had an unplanned pregnancy had lower mother-to-infant bonding quality, mostly prenatal (7 of 17, 4 of 9 and 4 of 10 studies respectively). Reported correlations were mostly weak. Some studies (4 of 10) examining the mode of birth showed that women who had a vaginal delivery had higher mother-to-infant bonding quality than women who had a cesarean section or elective labor induction.

#### Psychosocial

Mother-to-infant bonding quality measured at a certain time point during pregnancy or postpartum (earlier mother-to-infant bonding) was positively associated with mother-to-infant bonding quality measured later in time (prenatal or postnatal) in all 19 studies. Depression was operationalized in a variety of ways: In some studies, women were diagnosed according to DSM-IV criteria. Most studies, however, used depressive symptoms based on sum scores or cut-off points (or both) of self-report questionnaires like the Edinburg Postnatal Depression scale [33], which was most frequently used. The time of measurement of depressive symptoms varied between early pregnancy to 18 months postpartum. In almost all studies assessing the association between depressive symptoms and postnatal mother-to-infant bonding quality, negative associations were reported (28 of 29 studies): Women with more depressive symptoms, or categorized as depressed, had lower mother-to-infant bonding quality. Most correlations were moderate or strong. Studies examining prenatal bonding were less conclusive; a small majority of the studies (10 of 17) showed associations between prenatal depressive symptoms and lower mother-to-infant bonding quality. Social support was measured between the second trimester of pregnancy and 6 months postpartum. Only the Multidimensional Scale of Perceived Social Support [34] was used twice. In most studies sum scores were calculated. The majority of studies (8 of 13 studies) demonstrated positive associations of social support with mother-to-infant bonding quality, particularly prenatal (7 of 9). Correlations were mostly weak or moderate. Quality of partner relationship was measured with the Dyadic Adjustment Scale [35] in about one third of the studies and mostly sum scores were calculated. The time of measuring varied between early pregnancy and 6 months postpartum. A higher quality of partner relationship was positively associated with mother-to-infant bonding quality in 9 of 16 studies, with mostly weak correlations. General attachment (attachment to own caregivers or other peer adults) was measured with the Parental Bonding Instrument [36] or the Experiences in Close Relationships Inventory [37] in most studies. Mostly sum scores were calculated. Timing ranged between early pregnancy and 4 months postpartum. The few studies that did find associations (5 of 13) showed that stronger attachment to own caregivers, or more positive attachment styles (e.g. secure), were positively associated with mother-to-infant bonding quality. Correlations were weak or moderate. General anxiety symptoms were measured between the first trimester of pregnancy and 6 months postpartum, mostly with the State-Trait Anxiety Inventory [38] and the Hospital Anxiety and Depression Scale [39]. Both sum scores and dichotomized scores were used. Mixed results were found for the 5 studies examining prenatal bonding and the 8 studies examining postnatal bonding. Four studies found negative associations between level of general anxiety and postnatal bonding quality, 1 study found a positive association with postnatal mother-to-infant bonding quality. The other 8 studies found both negative and no associations, depending on measures used, timing of measures, and type of analysis applied. Correlations were mostly weak or moderate.

#### Child-related

The vast majority of studies that examined gender of the baby did not find an association with postnatal mother-to-infant bonding quality (9 of 11 studies). Two studies carried out in Bangladesh and in Turkey reported that having a boy was associated with higher mother-to-infant bonding quality.

## Discussion

### Main findings

Among 123 correlates of mother-to-infant bonding quality identified in this systematic review, 3 correlates (2 psychosocial and 1 reproduction-related) were consistently associated with mother-to-infant bonding quality: 1) prenatal mother-to-infant bonding quality was positively associated with duration of gestation at its assessment, 2) depressive symptoms were negatively associated with postnatal mother-to-infant bonding quality, and 3) mother-to-infant bonding quality earlier in pregnancy or in the postpartum period was positively associated with mother-to-infant bonding quality at later time points (both prenatal and postnatal). Further, desired pregnancy and social support were positively associated with mother-to-infant bonding quality in most studies. None of the demographic or child-related correlates were consistently associated with mother-to-infant bonding quality. Most child-related correlates of mother-to-infant bonding quality, however, were only examined in a few studies, making inferences less reliable. None of the partner-related correlates (e.g. paternal age, paternal stress) met our threshold of having been examined in at least 10 studies.

### Interpretation

In line with other reviews focusing on prenatal mother-to-infant bonding, we found limited evidence for an association between depressive symptoms and prenatal mother-to-infant bonding quality [19,20]. Interestingly, however, depressive symptoms were negatively associated with postnatal mother-to-infant bonding quality in the vast majority of studies. The negative association between depressive symptoms and mother-to-infant bonding quality might even be more pronounced once the baby is born since the development of a postnatal bond might also be affected by mother-baby interaction [40], a quality that might be compromised by maternal depressive symptoms [41]. Major clinical guidelines recommend universal screening and subsequent treatment of depression in the perinatal period [42,43]. The prevalence of postnatal depression is around 13% in the general population [44] and has been reported to be similar or even higher in the prenatal period [45–47]. Although more research on interventions aimed at preventing and reducing depressive symptoms is needed, there are several promising interventions aimed at both prenatal and postnatal depression [44,48,49]. Whether interventions aimed at reducing depressive symptoms might help to improve mother-to-infant bonding quality should be established in clinical trials. Furthermore, other maternal mental health factors such as general anxiety and stress have been examined considerably less often in relation to mother-to-infant bonding than depression, and yielded mixed findings. These factors deserve more attention in future research. General anxiety, for example, has been shown to be associated with poorer mother-infant interaction in several studies [50,51], which might in turn affect mother-to-infant bonding quality. Another gap of knowledge that should be addressed in future research is whether mental health prior to pregnancy is associated with mother-to-infant bonding quality. It has been found that episodes of pre-pregnancy depression is related to the trajectory of depressive symptoms during pregnancy [52].

In accordance with previous systematic reviews, we found consistent evidence for an association between duration of gestation at assessment and mother-to-infant bonding quality; as pregnancy progresses, mother-to-infant bonding quality increases [19,20]. This is consistent with the theory of Bowlby that suggests that the bond between child and caregiver increases naturally over time [53]. For clinical practice, this implies that a mother-to-infant bonding instrument to distinguish between disturbed and non-disturbed prenatal mother-to-infant bonding should take duration of gestation at assessment into account. Establishing cut-off points for each trimester of pregnancy might be appropriate.

Our finding that mother-to-infant bonding quality earlier in pregnancy or in the postpartum period is positively associated with mother-to-infant bonding quality later in time, both prenatally and postnatally, is not surprising. Nevertheless, it supports the theory that mother-to-infant bonding is a process that can start during pregnancy and continues after birth [1,4,5]. An implication of our finding is that monitoring of bonding should start already during pregnancy. However, more research on effective interventions aimed at improving mother-to-infant bonding is required as most research thus far yielded inconclusive results [54,55].

Concerning social support, which was found to be positively associated with prenatal bonding quality, our results correspond with the review by Yarcheski et al. [20]. According to Yarcheski et al. [20], social support might be especially important for more vulnerable populations, which were excluded in our review. The association between mother-to-infant bonding and other social factors (e.g. quality of the relationship with significant others beside the partner), which have been addressed only a few times, should be examined in future studies.

Our review showed a positive association between a desired pregnancy and prenatal bonding quality, whereas mixed findings were found for whether the pregnancy was planned. Presumably, women who feel more positive about their pregnancy also develop positive feelings towards their child more easily compared to women with an undesired pregnancy. An unplanned pregnancy, however, does not necessarily prevent the development of positive feelings towards the child, as un unplanned pregnancy can still be desired. This is in line with the results of studies which did not find whether the pregnancy was planned to be an important correlate of mother-to-infant bonding [20,22]. Therefore, we recommend future studies on bonding to address the degree to which a pregnancy is desired.

In accordance with the literature we did not find much evidence for an association between demographic correlates and mother-to-infant bonding quality either prenatally or postnatally [19,20]. As Yarcheski concludes, demographic factors do not seem to be important for clinical practice regarding prenatal mother-to-infant bonding [20]. Our study shows that this conclusion might concern postnatal mother-to-infant bonding as well. Partner-related and child-related factors were not discussed in previous reviews [19–22], and except for gender of the child, these were examined in only a few studies, making inferences difficult. More research is needed to find out if interventions aimed at improving mother-to-infant bonding quality should not only be aimed at the mother, but also at her child or partner.

Our findings inform further studies examining correlates of mother-to-infant bonding quality, and can be useful for maternal health care providers, psychologists, and pedagogical professionals. For example, awareness of maternal depression as an important threat to adequate mother-to-infant bonding may increase a maternal health care provider’s attention to signals of maternal depression, and may enable him or her to provide more effective care. To be able to understand the mechanisms behind mother-to-infant bonding and develop strategies to promote mother-to-infant bonding quality, however, more complex study designs including mediation and moderation analysis are needed. For example, knowing whether social support is important especially for more vulnerable populations, as suggested by Yarcheski et al [20], is important to develop effective interventions. In addition, we would recommend future reviews to focus on correlates of mother-to-infant bonding within specific subgroups (e.g. teenage pregnancies, mothers with preterm babies, mothers with lower income levels), which were not included in our systematic review.

### Strengths and limitations

To our knowledge, this is the first systematic review examining correlates of both prenatal and postnatal mother-to-infant bonding quality in the general population. To get a full picture of all possible correlates, we used broad search terms and did not put a limitation on the publication date. Including search terms referring to validated mother-to-infant bonding instruments included in our carefully composed list, further enhanced the reliability of our results. Furthermore, we performed reference checks of the included articles to decrease the chance that we failed to include relevant studies. The inter-observer variation of the screening of the titles and abstracts was good (kappa > .80) [56,57]. To enhance the quality of our systematic review and to enhance the transparency of our approach, we followed the PRISMA statement [27] for reporting our systematic review, and a study protocol was published beforehand in Prospero.

Some limitations should be taken into account when interpreting our findings. First, the quality of many (cross sectional) studies was poor. Second, the large heterogeneity of our included studies (e.g. in terms of measures used for mother-to-infant bonding, operationalization of the correlates, timing of correlates and outcomes, statistical analyses performed) might (partly) explain the inconsistent results and prevented drawing firm conclusions, let alone perform a meta-analysis. A third limitation might be that we only included published studies, and therefore there might have been publication bias [58] which we were not able to assess because we only conducted a narrative synthesis.

## Conclusions

Findings of our systematic review support the theory that mother-to-infant bonding is a process that starts during pregnancy and continues postnatally. Consequently, monitoring mother-to-infant bonding quality should already be considered during pregnancy. Interventions aimed at depressive symptoms, which were negatively associated with postnatal mother-to-infant bonding quality in the vast majority of studies, might help to improve mother-to-infant bonding. More high quality research on correlates for which mixed results were found (e.g. social support and a desired pregnancy), and correlates that were examined in only a few studies, is needed to guide the development of effective interventions.

## Supporting information

S1 File Search strategy in MEDLINE, Embase, CINAHL and PsychINFO. (DOCX)

S2 File Review protocol registered in PROSPERO (International prospective register of systematic reviews). (PDF)

S3 File Quality Assessment Tool for Observational Cohort and Cross-sectional studies of the National Heart, Lung, and Blood Institute. (DOCX)

S4 File References of all included studies. (DOCX)

S1 Table List of included measurements according to the definition of mother-to-infant bonding (the emotional or affective tie (feelings) experienced by a mother towards her child. (DOCX)

S2 Table Overview of included studies examining associations between demographic, reproduction-related, psychosocial, child-related and partner-related correlates and mother-to-infant bonding quality (prenatal and postnatal). (DOCX)

S3 Table Overview of all identified studies, identified by number, examining associations between correlates examined in less than 10 different studies with mother-to-infant bonding quality in the prenatal and postnatal period. (DOCX)

S4 Table PRISMA checklist. This checklist is showing various sections of this review and page numbers on which these sections are reported.
(DOC)
