# Supplementary material for: Correlates of prenatal and postnatal mother-to-infant bonding quality: A systematic review
Source: PLoS One. 2019 Sep 24;14(9):e0222998. doi: 10.1371/journal.pone.0222998 (PMC6759162; doi:10.1371/journal.pone.0222998)
Supplement: S1 File — (DOCX) [file pone.0222998.s001.docx]

**S1 File** Search strategy in MEDLINE, Embase, CINAHL and PsychINFO

MEDLINE
#1
("Pregnancy"[Mesh] OR pregnan*[tiab] OR gestation*[tiab] OR gravidit*[tiab] OR mother*[tiab] OR mom*[tiab] OR parent*[tiab]) AND (infant*[tiab] OR child*[tiab] OR toddler*[tiab] OR foetal[tiab] OR fetal[tiab] OR foetus[tiab] OR fetus[tiab] OR newborn*[tiab] OR baby[tiab] OR babies[tiab])
N= 490227
#2
("Object Attachment"[Mesh] OR "Parent-Child Relations"[Mesh] OR attachment[tiab] OR bond*[tiab])
N= 379352
#3
(“Maternal Antenatal Attachment Scale”[tiab] OR MAAS[tiab] OR “Maternal Attachment Inventory”[tiab] OR MAI[tiab] OR “Prenatal Attachment Inventory”[tiab] OR PAI[tiab] OR “Maternal Fetal Attachment Scale”[tiab] OR “Maternal Foetal Attachment Scale”[tiab] OR MFAS[tiab] OR “Maternal Postnatal Attachment Scale”[tiab] OR MPAS[tiab] OR “Maternal Postnatal Attachment Questionnaire”[tiab] OR MPAQ[tiab] OR “Mother-to-infant bonding scale” [tiab] OR MIB[tiab] OR MIBS[tiab] OR MIBQ[tiab] OR “Mother-Infant Bonding Scale”[tiab] OR “mother-to-infant bonding measure”[tiab] OR “Mother-Baby Bonding Scale”[tiab] OR “Postpartum Bonding Questionnaire” [tiab] OR PBQ[tiab] OR SPBQ[tiab] OR “Pregnancy involvement list”[tiab] OR PIL[tiab] OR “How I Feel About the Baby Now Scale”[tiab] OR FAB[tiab] OR HIFBN[tiab] OR “Antenatal Maternal Attachment Scale” [tiab] OR AMAS[tiab] OR “Modified Maternal Foetal Attachment Scale”[tiab] OR MMFAS[tiab])
N= 36813
#1 AND #2 AND #3

- 184 records identified on May 7th, 2018

Embase
#1
('pregnancy'/de OR pregnan*:ti OR pregnan*:ab OR gestation*:ti OR gestation*:ab OR gravidit*:ti OR gravidit*:ab OR mother*:ti OR mother*:ab OR mom*:ti OR mom*:ab OR parent*:ti OR parent*:ab) AND (infant*:ti OR infant*:ab OR child*:ti OR child*:ab OR toddler*:ti OR toddler*:ab OR foetal:ti OR foetal:ab OR fetal:ti OR fetal:ab OR foetus:ti OR foetus:ab OR fetus:ti OR fetus:ab OR newborn*:ti OR newborn*:ab OR baby:ti OR baby:ab OR babies:ti OR babies:ab)
N = 555,735
#2
('object relation'/de OR 'child parent relation'/de OR attachment:ti OR attachment:ab OR bond*:ti OR bond*:ab)
N= 352,054
#3
(‘Maternal Antenatal Attachment Scale’:ti OR ‘Maternal Antenatal Attachment Scale’:ab OR MAAS:ti OR MAAS:ab OR ‘Maternal Attachment Inventory’:ti OR ‘Maternal Attachment Inventory’:ab OR MAI:ti OR MAI:ab OR ‘Prenatal Attachment Inventory’:ti OR ‘Prenatal Attachment Inventory’:ab OR PAI:ti OR PAI:ab OR ‘Maternal Fetal Attachment Scale’:ti OR ‘Maternal Fetal Attachment Scale’:ab OR ‘Maternal Foetal Attachment Scale’:ti OR ‘Maternal Foetal Attachment Scale’:ab OR MFAS:ti OR MFAS:ab OR ‘Maternal Postnatal Attachment Scale’:ti OR ‘Maternal Postnatal Attachment Scale’:ab OR MPAS:ti OR MPAS:ab OR ‘Maternal Postnatal Attachment Questionnaire’:ti OR ‘Maternal Postnatal Attachment Questionnaire’:ab OR MPAQ:ti OR MPAQ:ab OR ‘Mother-to-infant bonding scale’:ti OR ‘Mother-to-infant bonding scale’:ab OR MIB:ti OR MIB:ab OR MIBS:ti OR MIBS:ab OR MIBQ:ti OR MIBQ:ab OR ‘Mother-Infant Bonding Scale’:ti OR ‘Mother-Infant Bonding Scale’:ab OR ‘mother-to-infant bonding measure’:ti OR ‘mother-to-infant bonding measure’:ab OR ‘Mother-Baby Bonding Scale’:ti OR ‘Mother-Baby Bonding Scale’:ab OR ‘Postpartum Bonding Questionnaire’:ti OR ‘Postpartum Bonding Questionnaire’:ab OR PBQ:ti OR PBQ:ab OR SPBQ:ti OR SPBQ:ab OR ‘Pregnancy involvement list’:ti OR ‘Pregnancy involvement list’:ab OR PIL:ti OR PIL:ab OR ‘How I Feel About the Baby Now Scale’:ti OR ‘How I Feel About the Baby Now Scale’:ab OR FAB:ti OR FAB:ab OR HIFBN:ti OR HIFBN:ab OR ‘Antenatal Maternal Attachment Scale’:ti OR ‘Antenatal Maternal Attachment Scale’:ab OR AMAS:ti OR AMAS:ab OR ‘Modified Maternal Foetal Attachment Scale’:ti OR ‘Modified Maternal Foetal Attachment Scale’:ab OR MMFAS:ti OR MMFAS:ab)
N = 48,694

- 270 records identified on May 7th, 2018

CINAHL
#1
(MH "Pregnancy" OR TI pregnan* OR AB pregnan* OR TI gestation* OR AB gestation* OR TI gravidit* OR AB gravidit* OR TI mother* OR AB mother* OR TI mom* OR AB mom* OR TI parent* OR AB parent*) AND (TI infant* OR AB infant* OR TI child* OR AB child* OR TI toddler* OR AB toddler* OR TI foetal OR AB foetal OR TI fetal OR AB fetal OR TI foetus OR AB foetus OR TI fetus OR AB fetus OR TI newborn* OR AB newborn* OR TI baby OR AB baby OR TI babies OR AB babies)
N = 85,708
#2
AND (MH “Attachment Behavior" OR MH “Parent-Child Relations" OR TI attachment OR AB attachment OR TI bond* OR AB bond*)
N= 18,750
#3
AND (TI “Maternal Antenatal Attachment Scale” OR AB “Maternal Antenatal Attachment Scale” OR TI MAAS OR AB MAAS OR TI “Maternal Attachment Inventory” OR AB “Maternal Attachment Inventory” OR TI MAI OR AB MAI OR TI “Prenatal Attachment Inventory” OR AB “Prenatal Attachment Inventory” OR TI PAI OR AB PAI OR TI “Maternal Fetal Attachment Scale” OR AB “Maternal Fetal Attachment Scale” OR TI “Maternal Foetal Attachment Scale” OR AB “Maternal Foetal Attachment Scale” OR TI MFAS OR AB MFAS OR TI “Maternal Postnatal Attachment Scale” OR AB “Maternal Postnatal Attachment Scale” OR TI MPAS OR AB MPAS OR TI “Maternal Postnatal Attachment Questionnaire” OR AB “Maternal Postnatal Attachment Questionnaire” OR TI MPAQ OR AB MPAQ OR TI “Mother-to-infant bonding scale” OR AB “Mother-to-infant bonding scale” OR TI MIB OR AB MIB OR TI MIBS OR AB MIBS OR TI MIBQ OR AB MIBQ OR TI “Mother-Infant Bonding Scale” OR AB “Mother-Infant Bonding Scale” OR TI “mother-to-infant bonding measure” OR AB “mother-to-infant bonding measure” OR TI “Mother-Baby Bonding Scale” OR AB “Mother-Baby Bonding Scale” OR TI “Postpartum Bonding Questionnaire” OR AB “Postpartum Bonding Questionnaire” OR TI PBQ OR AB PBQ OR TI SPBQ OR AB SPBQ OR TI “Pregnancy involvement list” OR AB “Pregnancy involvement list” OR TI PIL OR AB PIL OR TI “How I Feel About the Baby Now Scale” OR AB “How I Feel About the Baby Now Scale” OR TI FAB OR AB FAB OR TI HIFBN OR AB HIFBN OR TI “Antenatal Maternal Attachment Scale” OR AB “Antenatal Maternal Attachment Scale” OR TI AMAS OR AB AMAS OR TI “Modified Maternal Foetal Attachment Scale” OR AB “Modified Maternal Foetal Attachment Scale” OR TI MMFAS OR AB MMFAS)
N = 2,675

- 137 records identified on May 7th, 2018

PsychINFO
(DE "Pregnancy" OR TI pregnan* OR AB pregnan* OR TI gestation* OR AB gestation* OR TI gravidit* OR AB gravidit* OR TI mother* OR AB mother* OR TI mom* OR AB mom* OR TI parent* OR AB parent*) AND (TI infant* OR AB infant* OR TI child* OR AB child* OR TI toddler* OR AB toddler* OR TI foetal OR AB foetal OR TI fetal OR AB fetal OR TI foetus OR AB foetus OR TI fetus OR AB fetus OR TI newborn* OR AB newborn* OR TI baby OR AB baby OR TI babies OR AB babies)
N= 211,055
AND (DE “Attachment Behavior" OR DE “Parent Child Relations" OR TI attachment OR AB attachment OR TI bond* OR AB bond*)
N= 68,514
(TI “Maternal Antenatal Attachment Scale” OR AB “Maternal Antenatal Attachment Scale” OR TI MAAS OR AB MAAS OR TI “Maternal Attachment Inventory” OR AB “Maternal Attachment Inventory” OR TI MAI OR AB MAI OR TI “Prenatal Attachment Inventory” OR AB “Prenatal Attachment Inventory” OR TI PAI OR AB PAI OR TI “Maternal Fetal Attachment Scale” OR AB “Maternal Fetal Attachment Scale” OR TI “Maternal Foetal Attachment Scale” OR AB “Maternal Foetal Attachment Scale” OR TI MFAS OR AB MFAS OR TI “Maternal Postnatal Attachment Scale” OR AB “Maternal Postnatal Attachment Scale” OR TI MPAS OR AB MPAS OR TI “Maternal Postnatal Attachment Questionnaire” OR AB “Maternal Postnatal Attachment Questionnaire” OR TI MPAQ OR AB MPAQ OR TI “Mother-to-infant bonding scale” OR AB “Mother-to-infant bonding scale” OR TI MIB OR AB MIB OR TI MIBS OR AB MIBS OR TI MIBQ OR AB MIBQ OR TI “Mother-Infant Bonding Scale” OR AB “Mother-Infant Bonding Scale” OR TI “mother-to-infant bonding measure” OR AB “mother-to-infant bonding measure” OR TI “Mother-Baby Bonding Scale” OR AB “Mother-Baby Bonding Scale” OR TI “Postpartum Bonding Questionnaire” OR AB “Postpartum Bonding Questionnaire” OR TI PBQ OR AB PBQ OR TI SPBQ OR AB SPBQ OR TI “Pregnancy involvement list” OR AB “Pregnancy involvement list” OR TI PIL OR AB PIL OR TI “How I Feel About the Baby Now Scale” OR AB “How I Feel About the Baby Now Scale” OR TI FAB OR AB FAB OR TI HIFBN OR AB HIFBN OR TI “Antenatal Maternal Attachment Scale” OR AB “Antenatal Maternal Attachment Scale” OR TI AMAS OR AB AMAS OR TI “Modified Maternal Foetal Attachment Scale” OR AB “Modified Maternal Foetal Attachment Scale” OR TI MMFAS OR AB MMFAS)
N= 3,829

- 306 records identified on May 7th, 2018
