# Supplementary material for: Correlates of prenatal and postnatal mother-to-infant bonding quality: A systematic review
Source: PLoS One. 2019 Sep 24;14(9):e0222998. doi: 10.1371/journal.pone.0222998 (PMC6759162; doi:10.1371/journal.pone.0222998)
Supplement: S2 File — (PDF) [file pone.0222998.s002.pdf]

S2 File. **Review protocol registered in PROSPERO** (International prospective register of systematic reviews).

PROSPERO International prospective register of systematic reviews

Review title and timescale

1 Review title
Give the working title of the review. This must be in English. Ideally it should state succinctly the interventions or exposures being reviewed and the associated health or social problem being addressed in the review.
Predictors of prenatal and postnatal mother-to-infant bonding: A systematic review.

2 Original language title
For reviews in languages other than English, this field should be used to enter the title in the language of the review. This will be displayed together with the English language title.

3 Anticipated or actual start date
Give the date when the systematic review commenced, or is expected to commence.
10/06/2016

4 Anticipated completion date
Give the date by which the review is expected to be completed.
31/12/2016

5 Stage of review at time of this submission
Indicate the stage of progress of the review by ticking the relevant boxes. Reviews that have progressed beyond the point of completing data extraction at the time of initial registration are not eligible for inclusion in PROSPERO. This field should be updated when any amendments are made to a published record.

The review has not yet started ×

Review stage	Started	Completed
Preliminary searches	Yes	No
Piloting of the study selection process	No	No
Formal screening of search results against eligibility criteria	No	No
Data extraction	No	No
Risk of bias (quality) assessment	No	No
Data analysis	No	No

Provide any other relevant information about the stage of the review here.
final protocol finalised

Review team details

6 Named contact
The named contact acts as the guarantor for the accuracy of the information presented in the register record.
Elke Tichelman

7 Named contact email
Enter the electronic mail address of the named contact.
e.tichelman@umcg.nl

8 Named contact address
Enter the full postal address for the named contact.
Midwifery Science AVAG EMGO+ institute for Health and Care Research| VU Medical Center Amsterdam Van der Boechorstraat 7 (room A-505) | 1081 BT Amsterdam The Netherlands

9 Named contact phone number
Enter the telephone number for the named contact, including international dialing code.
0031-20-4446446

10 Organisational affiliation of the review
Full title of the organisational affiliations for this review, and website address if available. This field may be completed

as 'None' if the review is not affiliated to any organisation.
Midwifery Science AVAG EMGO+ institute for Health and Care Research|

Website address:

11 Review team members and their organisational affiliations
Give the title, first name and last name of all members of the team working directly on the review. Give the organisational affiliations of each member of the review team.

Title	First name	Last name	Affiliation
Ms	Elke	Tichelman	Midwifery Science AVAG EMGO+ institute for Health and Care Research|, Department of General practice, University Medical Centre Groningen, the Netherlands
Ms	Myrte	Westerneng	Department of Midwifery Science, AVAG EMGO+ Institute for Health and Care Research, VU University Medical Centre, The Netherlands
Dr	Anke	Witteveen	Department of Midwifery Science, AVAG EMGO+ Institute for Health and Care Research, VU University Medical Centre, The Netherlands
Dr	A.L.	van Baar	Child and Adolescent Studies, Utrecht University, the Netherlands
Dr	H.E.	van der Horst	Department of General Practice, VU University Medical Center EMGO Institute, VU University Medical Center
Dr	A.	de Jonge	Department of Midwifery Science, AVAG EMGO+ Institute for Health and Care Research, VU University Medical Centre, The Netherlands
Dr	M.Y.	Berger	Department of General practice, University Medical Centre Groningen, the Netherlands
Dr	F.G.	Schellevis	Department of Midwifery Science, AVAG EMGO+ Institute for Health and Care Research, VU University Medical Centre, The Netherlands
Dr	H.	Burger	Department of General practice, University Medical Centre Groningen, the Netherlands
Dr	Lilian	Peters	Department of Midwifery Science, AVAG EMGO+ Institute for Health and Care Research, VU University Medical Centre, The Netherlands

12 Funding sources/sponsors
Give details of the individuals, organizations, groups or other legal entities who take responsibility for initiating, managing, sponsoring and/or financing the review. Any unique identification numbers assigned to the review by the individuals or bodies listed should be included.
Department of Midwifery Science, AVAG EMGO+ Institute for Health and Care Research, VU University Medical Centre, The Netherlands Department of General practice, University Medical Centre Groningen, the Netherlands

13 Conflicts of interest
List any conditions that could lead to actual or perceived undue influence on judgements concerning the main topic investigated in the review.
Are there any actual or potential conflicts of interest?
None known

14 Collaborators
Give the name, affiliation and role of any individuals or organisations who are working on the review but who are not

listed as review team members.

Title	First name	Last name	Organisation details

Review methods

15 Review question(s)

State the question(s) to be addressed / review objectives. Please complete a separate box for each question.

Which maternal, child, and contextual factors predict the level of prenatal and postnatal mother-to-infant bonding up to early childhood?

16 Searches

Give details of the sources to be searched, and any restrictions (e.g. language or publication period). The full search strategy is not required, but may be supplied as a link or attachment.

Relevant studies will be identified by searching PsychINFO, MEDLINE, Embase, and CINAHL up till June 2016. The searches will be re-run in the summer of 2016 just before the final analyses to make sure our search is as up-to-date as possible. The search will be assisted by a librarian. We will also search for unpublished literature, dissertations and conference proceedings and include additional studies retrieved by inspection of reference lists of relevant studies and reviews, and additional studies obtained by contacted authors and other experts. Search terms related to the population (pregnancy, mother, infant) will be used in our search, as well as search terms related to our outcome (mother-to-infant bonding). Additionally, we will include names of measurements in our search that measure mother-to-infant bonding according to the definition of Kinsey et al. (2011). To be able to include all relevant measurements, we first performed a search with search terms related to the population and outcome as described above, supplemented with search terms related to measurement tools (e.g. surveys, questionnaires, scale). Subsequently, two researchers each scanned the first 1000 hits comprising the most recent articles in order to extract relevant measurement instruments from these studies . Furthermore, well-known articles providing an overview of mother-to-infant bonding were checked (Perelli et al., 2014; van den Bergh et al., 2009). This resulted in the following search strategy for Pubmed: (see the attached pdf). Search terms will be adjusted for use in the other databases. Full copies of papers have to be written in English, German, Swedish, Norwegian, French or Spanish or Dutch. Duplicate studies will be removed.

17 URL to search strategy

If you have one, give the link to your search strategy here. Alternatively you can e-mail this to PROSPERO and we will store and link to it.

http://www.crd.york.ac.uk/PROSPEROFILES/40183_STRATEGY_20160510.pdf

I give permission for this file to be made publicly available

No

18 Condition or domain being studied

Give a short description of the disease, condition or healthcare domain being studied. This could include health and wellbeing outcomes.

mother-to-infant bonding, predictors

19 Participants/population

Give summary criteria for the participants or populations being studied by the review. The preferred format includes details of both inclusion and exclusion criteria.

Pregnant women or biological mothers of children up till early childhood age will be included. Non-humans, fathers and adoptive mothers will be excluded, as well as specific subgroups of mothers (e.g. with teenage pregnancies, preterm children, intimate partner violence, psychiatric diagnoses, or HIV, surrogate mothers, substance-abusing mothers, blind or deaf mothers, mothers of a diseased child) which do not contrast these groups with the general population.

20 Intervention(s), exposure(s)

Give full and clear descriptions of the nature of the interventions or the exposures to be reviewed

Factors suggested to predict mother-to-infant bonding are maternal factors (e.g. mental health), child factors (e.g. gestational age at birth), contextual factors (e.g. partner support) (de Cock et al., 2015) and biological factors (Atzil et al., 2011; Galbally et al, 2011; Kendrick et al., 2000). To give a complete overview of factors, however, we do not limit our search based on these known types of factors and do not exclude any factors in advance. We do exclude factors which are clearly not a predictor of mother-to-infant bonding, but a result (e.g. developmental outcomes of the child

associated with prenatal mother to-infant bonding). Factors that might be an outcome as well as a predictor of mother-to-infant bonding (e.g. oxytocin) will be included.

21 Comparator(s)/control

Where relevant, give details of the alternatives against which the main subject/topic of the review will be compared (e.g. another intervention or a non-exposed control group).
Absence or reduced level of the risk factor

22 Types of study to be included initially

Give details of the study designs to be included in the review. If there are no restrictions on the types of study design eligible for inclusion, this should be stated.
Epidemiologic studies that assess determinants/risk factors such as, case-control studies, cohort studies, cross-sectional and longitudinal studies using both prospective and retrospective designs will be included. Experimental studies will be excluded . Also Qualitative studies and studies that provided no new empirical data (reviews and editorial letters) will be excluded.

23 Context

Give summary details of the setting and other relevant characteristics which help define the inclusion or exclusion criteria.
We will include studies carried out in any setting.

24 Primary outcome(s)

Give the most important outcomes.
We consider feelings of a mother towards her child as the most important indicator of mother-to-infant bonding . Consequently, this systematic review focuses on studies which operationalize mother-to-infant bonding through self-reported feelings of the mother towards her child, either by a questionnaire or interview. Studies that operationalize mother-to-infant bonding solely as behaviors of a mother towards her child are not considered eligible . Furthermore, we will exclude studies that use measurements that approach attachment or bonding from the (partially) perspective of the child or from an interactive perspective. Examples of such measurements are the strange situation test and the Attachment Q-set (AQS). Studies using measurements which focus on attachment patterns of the mother in general and not specific from mother to child will also be excluded (e.g. the Adult Attachment Interview; AAI). Finally, studies using measurements that focus on concepts related to mother-to-infant bonding but do not meet our definition since their primary focus is not on feelings of the mother towards her child, will be excluded. Examples of these are the Working Mother of the Child interview (WMCI), a measurement focusing on maternal representations of the child and the Maternal Adjustment and Maternal Attitudes (MAMA) scale, a measurement focusing on adjustment and attitude of a woman during her pregnancy. We also excluded the Pictorial Representation of Attachment Measure (PRAM; van Bakel, 2009). Although it is intended to measure the bond between the mother and her unborn child, it asks women where they would place their baby in their life at the moment. This approach does not directly reflect the affective component that we see as the primary indicator of bonding. Furthermore, measurements focusing on parental sensitivity (mother's ability to perceive and infer the meaning behind her infant's behaviour signals and to respond to them promptly and appropriately, e.g. Ainsworth's Maternal Sensitivity Scale; AMSS) or mindful parenting (e.g. Interpersonal Mindfulness in Parenting scale; IM-P) will also be excluded.

Give information on timing and effect measures, as appropriate.

25 Secondary outcomes

List any additional outcomes that will be addressed. If there are no secondary outcomes enter None.
none

Give information on timing and effect measures, as appropriate.

26 Data extraction, (selection and coding)

Give the procedure for selecting studies for the review and extracting data, including the number of researchers involved and how discrepancies will be resolved. List the data to be extracted.
Two researchers (ET and MW) will independently screen titles and abstracts. In case of uncertainties or disagreement a third researcher? will be consulted (ABW). The same screening procedure will be applied to full texts retrieved for all studies included based on title and abstract. An electronic link to the list of included and excluded studies will be provided. For those studies that are included, MW and ET will independently extract data using a previously prepared data extraction form. From each of the included articles, at least the following information will be retrieved: first

author's name, publication year, study site and setting, study design, sample size, sample characteristics, outcome (prenatal or postnatal mother-to-infant bonding), examined predictors, measurements used for predictors and outcomes, confounding factors controlled for. If data allow it, information on the effect estimation (mean difference/OR/RR) and variance (SE) will be extracted. Any difference in extracted data between completed forms will be resolved by re-reviewing the corresponding articles and reaching consensus agreement. The PRISMA guidelines for the presentation of systematic reviews will be followed. This study will provide a flow diagram of information through the different phases of this systematic review (identification, screening, full text articles assessed for eligibility).

27 Risk of bias (quality) assessment

State whether and how risk of bias will be assessed, how the quality of individual studies will be assessed, and whether and how this will influence the planned synthesis.

MW and ET will assess the quality of each included study using a risk of bias tool called The Newcastle-Ottawa Scale (NOS). Each included study will get a description of quality items with a score of low or high risk of bias.

28 Strategy for data synthesis

Give the planned general approach to be used, for example whether the data to be used will be aggregate or at the level of individual participants, and whether a quantitative or narrative (descriptive) synthesis is planned. Where appropriate a brief outline of analytic approach should be given.

Clinical and methodological heterogeneity of the studies will be assessed and in case they are sufficiently comparable, statistical heterogeneity will be evaluated and data will be meta-analyzed with RevMan. Effect estimates and the weight of each study will be entered. For dichotomous data, we will present results as an adjusted odds ratio (OR), adjusted relative risk ratio(RR), adjusted absolute relative risk reduction (ARR) or adjusted hazard ratio (HR) with 95% con?dence intervals (CI). For continuous data, we will use the mean difference(MD) if outcomes are measured in the same way between trials. We will use the standardized mean difference (SMD) to combine trials that measure the same outcome, but uses different methods. Statistical approaches described in the Cochrane handbook for combining dichotomous outcomes and continuous outcomes will be used. The appropriateness of combining study results will be taken into consideration by examining the variety in methodological and clinical characteristics of the included studies. If methodological and clinical characteristics of the included studies are too different, the evidence will be summarized narratively. If not, heterogeneity will be quantified by the Q-test and the I2 value. Based on the results,a meta-analysis will be performed by using a random or fixed effects model.

29 Analysis of subgroups or subsets

Give any planned exploration of subgroups or subsets within the review. 'None planned' is a valid response if no subgroup analyses are planned.

If the data allow it, we will conduct subgroup analysis by dividing the outcome in prenatal and postnatal mother-to-infant bonding. Other subgroup analysis may be performed based on sources of heterogeneity appearing from our search (e.g. used measurements of mother-to-infant bonding). If necessary sensitivity analyses will be performed.

Review general information

30 Type of review

Select the type of review from the drop down list.

Epidemiologic

31 Language

Select the language(s) in which the review is being written and will be made available, from the drop down list. Use the control key to select more than one language.

English

Will a summary/abstract be made available in English?

Yes

32 Country

Select the country in which the review is being carried out from the drop down list. For multi-national collaborations select all the countries involved. Use the control key to select more than one country.

Netherlands

33 Other registration details

Give the name of any organisation where the systematic review title or protocol is registered together with any unique

identification number assigned. If extracted data will be stored and made available through a repository such as the Systematic Review Data Repository (SRDR), details and a link should be included here.

34 Reference and/or URL for published protocol

Give the citation for the published protocol, if there is one.
Give the link to the published protocol, if there is one. This may be to an external site or to a protocol deposited with CRD in pdf format.

http://www.crd.york.ac.uk/PROSPEROFILES/40183_PROTOCOL_20160510.pdf

I give permission for this file to be made publicly available

No

35 Dissemination plans

Give brief details of plans for communicating essential messages from the review to the appropriate audiences.
Do you intend to publish the review on completion?

Yes

36 Keywords

Give words or phrases that best describe the review. (One word per box, create a new box for each term)

parent child relations

bonding

attachment

pregnancy

predictors

37 Details of any existing review of the same topic by the same authors

Give details of earlier versions of the systematic review if an update of an existing review is being registered, including full bibliographic reference if possible.

38 Current review status

Review status should be updated when the review is completed and when it is published.

Ongoing

39 Any additional information

Provide any further information the review team consider relevant to the registration of the review.

40 Details of final report/publication(s)

This field should be left empty until details of the completed review are available.
Give the full citation for the final report or publication of the systematic review.
Give the URL where available.
