# Supplementary material for: Correlates of prenatal and postnatal mother-to-infant bonding quality: A systematic review
Source: PLoS One. 2019 Sep 24;14(9):e0222998. doi: 10.1371/journal.pone.0222998 (PMC6759162; doi:10.1371/journal.pone.0222998)
Supplement: S4 File — (DOCX) [file pone.0222998.s004.docx]

**S4 File** References of all included studies

1. Abuhammad SH. The possible effect of breastfeeding on maternal sensitivity during the first year of life. 2016.

2. Alan H, Ege E. The influence of social support on maternal-infant attachment in Turkish society. J Anatolia Nurs Heal Sci. 2013;16:234–40.

3. Andrek A, Kekecs Z, Hadhazi E, Boukydis Z, Varga K. Re-Evaluation of the Psychometric Properties of the Maternal–Fetal Attachment Scale in a Hungarian Sample. J Obstet Gynecol Neonatal Nurs. 2016;45:e15–e25.

4. Armstrong D, Hutti M. Pregnancy after perinatal loss: the relationship between anxiety and prenatal attachment. J Obstet Gynecol Neonatal Nurs. 1998;27:183–89.

5. Barone L, Lionetti F, Dellagiulia A. Maternal-fetal attachment and its correlates in a sample of Italian women: a study using the Prenatal Attachment Inventory. J Reprod Infant Psychol. 2014;32:230–39.

6. Bennington LK. The Relationship Among Maternal–Infant Bonding, Spirituality, and Maternal Perception of the Childbirth Experience. J Obstet Gynecol Neonatal Nurs. 2012;41:S137-S137.

7. Berryman JC, Windridge KC. Pregnancy after 35 and attachment to the fetus. J Reprod Infant Psychol. 1996;14:133–43.

8. Bicking Kinsey C. Effect of Prior Perinatal Loss on Maternal Depressive Symptoms and Maternal-Infant Bonding. The Pennsylvania State University; 2014.

9. Bielawska‐Batorowicz E, Siddiqui A. A study of prenatal attachment with Swedish and Polish expectant mothers. J Reprod Infant Psychol. 2008;26:373–84.

10. Boztepe H, Ay A, Kerimoğlu Yıldız G, Çınar S. Does the visibility of a congenital anomaly affect maternal–infant attachment levels? J Spec Pediatr Nurs. 2016;21:200–11.

11. Busonera A, Cataudella S, Lampis J, Tommasi M, Zavattini GC. Psychometric properties of a 20-item version of the Maternal–Fetal Attachment Scale in a sample of Italian expectant women. Midwifery. 2016;34:79–87.

12. Van Bussel JCH, Spitz B, Demyttenaere K. Three self-report questionnaires of the early mother-to-infant bond: reliability and validity of the Dutch version of the MPAS, PBQ and MIBS. Arch Womens Ment Health. 2010;13:373–84.

13. Calhoun AM. Impact of Skin-to-Skin Contact after Cesarean Section: A Study Comparing Bonding Outcomes to Skin-to-Skin after Vaginal Delivery. 2015.

14. Cetisli NE, Arkan G, Top ED. Maternal attachment and breastfeeding behaviors according to type of delivery in the immediate postpartum period. Rev Assoc Med Bras. 2018;64:164–69.

15. Chang H-C, Chen S-Y, Chen C-H. Predictors of Antenatal Psychosocial Stress in Taiwanese Women. J Nurs Res. 2016;24:193–200.

16. Chazotte C, Freda MC, Elovits M, Youchah J. Maternal depressive symptoms and maternal–fetal attachment in gestational diabetes. J Women’s Heal. 1995;4:375–80.

17. Chen C, Chen Y, Sung H, Kuo P, Wang C. Perinatal attachment in naturally pregnant and infertility‐treated pregnant women in Taiwan. J Adv Nurs. 2011;67:2200–08.

18. Cinar N, Köse D, Altinkaynak S. The relationship between maternal attachment, perceived social support and breast-feeding sufficiency. J Coll Physicians Surg Pakistan. 2015;25:271–75.

19. de Cock ESA, Henrichs J, Klimstra TA, et al. Longitudinal associations between parental bonding, parenting stress, and executive functioning in toddlerhood. J Child Fam Stud. 2017;26:1723–33.

20. Condon JT, Corkindale C. The correlates of antenatal attachment in pregnant women. Psychol Psychother Theory, Res Pract. 1997;70:359–72.

21. Cranley MS. Development of a tool for the measurement of maternal attachment during pregnancy. Nurs Res. 1981;30:281–84.

22. Cranley MS. Social support as a factor in the development of parents’ attachment to their unborn. Birth Defects Orig Artic Ser. 1984;20:99–124.

23. Delavari M, Mirghafourvand M, Mohammad-Alizadeh-Charandabi S. The relationship of maternal–fetal attachment and depression with social support in pregnant women referring to health centers of Tabriz–Iran, 2016. J Matern Neonatal Med. 2018;31:2450–56.

24. Della Vedova AM, Dabrassi F, Imbasciati A. Assessing prenatal attachment in a sample of Italian women. J Reprod Infant Psychol. 2008;26:86–98.

25. Della Vedova AM, Ducceschi B, Cesana BM, Imbasciati A. Maternal bonding and risk of depression in late pregnancy: a survey of Italian nulliparous women. J Reprod Infant Psychol. 2011;29:208–22.

26. Denis A, Callahan S, Bouvard M. Examination of the psychometric properties of the French translation of the Maternal Antenatal Attachment Scale (MAAS). Encephale. 2015;41:32–38.

27. Doster A, Wallwiener S, Müller M, et al. Reliability and validity of the German version of the Maternal–Fetal Attachment Scale. Arch Gynecol Obstet. 2018;297:1157–67.

28. Dubber S, Reck C, Müller M, Gawlik S. Postpartum bonding: the role of perinatal depression, anxiety and maternal–fetal bonding during pregnancy. Arch Womens Ment Health. 2015;18:187–95.

29. Edhborg M, Matthiesen AS, Lundh W, Widström AM. Some early indicators for depressive symptoms and bonding 2 months postpartum–a study of new mothers and fathers. Arch Womens Ment Health. 2005;8:221–31.

30. Edhborg M, Nasreen H-E, Kabir ZN. Impact of postpartum depressive and anxiety symptoms on mothers’ emotional tie to their infants 2–3 months postpartum: a population-based study from rural Bangladesh. Arch Womens Ment Health. 2011;14:307-16.

31. Feldstein S, Hane AA, Morrison BM, Huang K. Relation of the Postnatal Attachment Questionnaire to the Attachment Q‐Set. J Reprod Infant Psychol. 2004;22:111–21.

32. Figueiredo B, Costa R, Pacheco A, Conde A, Teixeira C. Anxiété, dépression et investissement émotionnel de l’enfant pendant la grossesse. Devenir. 2007;19:243–260.

33. Figueiredo B, Costa R, Pacheco A, Pais Á. Mother-to-infant emotional involvement at birth. Matern Child Health J. 2009;13:539–49.

34. Foster SF, Slade P, Wilson K. Body image, maternal fetal attachment, and breast feeding. J Psychosom Res. 1996;41:181­–84.

35. Friedman KB. Are symptoms of postpartum depression associated with deficits in facial and auditory emotional recognition? 2008.

36. Fuller SG, Moore LR, Lester JW. Influence of family functioning on maternal-fetal attachment. J Perinatol Off J Calif Perinat Assoc. 1993;13:453–60.

37. Gaffney KF. Maternal–fetal attachment in relation to self-concept and anxiety. Matern Child Nurs J. 1986;15:91–101

38. Garcia-Esteve L, Torres A, Lasheras G, et al. Assessment of psychometric properties of the Postpartum Bonding Questionnaire (PBQ) in Spanish mothers. Arch Womens Ment Health. 2016;19:385–94.

39. Gau M-L. The relationships between prenatal stress, social support, spiritual well-being, and maternal-fetal attachment for pregnant women. 1996.

40. Gaudet C, Séjourné N, Camborieux L, Rogers R, Chabrol H. Pregnancy after perinatal loss: Association of grief, anxiety and attachment. J Reprod Infant Psychol. 2010;28:240–51.

41. Gharaibeh MK, Hamlan AM. Factors influencing maternal attachment of first-time Jordanian mothers. J Res Nurs. 2012;17:289–303.

42. Goecke TW, Voigt F, Faschingbauer F, Spangler G, Beckmann MW, Beetz A. The association of prenatal attachment and perinatal factors with pre-and postpartum depression in first-time mothers. Arch Gynecol Obstet. 2012;286:309-16.

43. Grace JT. Development of maternal-fetal attachment during pregnancy. Nurs Res. 1989;38:228–32.

44. Haedt A, Keel P. Maternal attachment, depression, and body dissatisfaction in pregnant women. J Reprod Infant Psychol. 2007;25:285–95.

45. Hairston IS, Waxler E, Seng JS, Fezzey AG, Rosenblum KL, Muzik M. The role of infant sleep in intergenerational transmission of trauma. Sleep. 2011;34:1373–83.

46. Hall RAS, Hoffenkamp HN, Tooten A, Braeken J, Vingerhoets AJJM, Van Bakel HJA. Child-rearing history and emotional bonding in parents of preterm and full-term infants. J Child Fam Stud. 2015;24:1715–26.

47. Handelzalts JE, Krissi H, Levy S, et al. Personality, preterm labor contractions, and psychological consequences. Arch Gynecol Obstet. 2016;293:575–82.

48. Harpel T. Pregnant Women Sharing Pregnancy-Related Information on Facebook: Web-Based Survey Study. J Med Internet Res. 2018;20:e115.

49. Herguner S, Çiçek E, Annagur A, Herguner A, Ors R. Association of Delivery Type with Postpartum Depression, Perceived Social Support and Maternal Attachment/Dogum seklinin dogum sonrasi depresyon, algilanan sosyal destek ve maternal baglanma ile iliskisi. Dusunen Adam. 2014;27:15–20.

50. Himani BK, Kumar P. Effect of initiation of breastfeeding within one hour of the delivery on" maternal-infant bonding. Nurs Midwifery Res J. 2011;7:99–109.

51. Hjelmstedt A, Widström A, Collins A. Psychological correlates of prenatal attachment in women who conceived after in vitro fertilization and women who conceived naturally. Birth. 2006;33:303–10.

52. Høivik MS, Burkeland NA, Linaker OM, Berg‐Nielsen TS. The Mother and Baby Interaction Scale: a valid broadband instrument for efficient screening of postpartum interaction? A preliminary validation in a Norwegian community sample. Scand J Caring Sci. 2013;27:733–39.

53. Honjo S, Arai S, Kaneko H, et al. Antenatal depression and maternal-fetal attachment. Psychopathology. 2003;36:304–11.

54. Horsch A, Jacobs I, Gilbert L, et al. Impact of perinatal asphyxia on parental mental health and bonding with the infant: a questionnaire survey of Swiss parents. BMJ Paediatr Open. 2017;1:e000059.

55. Hsu T-L, Chen C-H. Stress and maternal-fetal attachment of pregnant women during their third trimester. Kaohsiung J Med Sci. 2001;17:36–45.

56. Kaminer AD. Life events stress, social support, and maternal-fetal attachment in incarcerated pregnant women and non-incarcerated pregnant women. 1992.

57. Kaneko H, Honjo S. The psychometric properties and factor structure of the Postpartum Bonding Questionnaire in Japanese mothers. Psychology. 2014;5:1135–42.

58. Kemp VH, Page CK. Maternal prenatal attachment in normal and high‐risk pregnancies. J Obstet Gynecol Neonatal Nurs. 1987;16:179–84.

59. Kita S, Haruna M, Matsuzaki M, Kamibeppu K. Associations between intimate partner violence (IPV) during pregnancy, mother-to-infant bonding failure, and postnatal depressive symptoms. Arch Womens Ment Health. 2016;19:623–34.

60. Kokubu M, Okano T, Sugiyama T. Postnatal depression, maternal bonding failure, and negative attitudes towards pregnancy: a longitudinal study of pregnant women in Japan. Arch Womens Ment Health. 2012;15:211–16.

61. Kraft A, Knappe S, Petrowski K, Petzoldt J, Martini J. Maternal bonding and infant attachment in women with and without social phobia. Z Kinder Jugendpsychiatr Psychother. 2017;45:49–57.

62. Krisjanous J, Richard JE, Gazley A. The Perfect Little Bump: Does the Media Portrayal of Pregnant Celebrities Influence Prenatal Attachment? Psychol Mark. 2014;31:758–73.

63. Kunkel GF. Fetal attachment and depression: measurement matters. J Prenat Perinat Psychol Heal. 2003;18:149–66.

64. Lahann RL. An examination of maternal-fetal attachment in singleton and twin pregnancies. 2008.

65. Lear TV. An Exploration of Maternal Prenatal Attachment as It Relates to Breastfeeding Intentions. 2013.

66. Lee TY. Maternal-fetal attachment in normal and previously infertile Chinese women. Nurs Res. 1994;2:67–77.

67. Lerum CW, LoBiondo‐Wood G. The Relationship of Maternal Age, Quickening, and Physical Symptoms of Pregnancy to the Development of Maternal‐Fetal Attachment. Birth. 1989;16:13–17.

68. Levine A, Zagoory-Sharon O, Feldman R, Weller A. Oxytocin during pregnancy and early postpartum: individual patterns and maternal–fetal attachment. Peptides. 2007;28:1162–69.

69. Lindgren K. Relationships among maternal–fetal attachment, prenatal depression, and health practices in pregnancy. Res Nurs Health. 2001;24:203–17.

70. Lingeswaran A, Bindu H. Validation of Tamil Version of Cranley’s 24-Item Maternal–Fetal Attachment Scale in Indian Pregnant Women. J Obstet Gynecol India. 2012;62:630–34.

71. Mason ZS. The role of maternal attachment: Its effect on postpartum depression and infant social-emotional development. 2009.

72. Mazzeschi C, Pazzagli C, Radi G, Raspa V, Buratta L. Antecedents of maternal parenting stress: the role of attachment style, prenatal attachment, and dyadic adjustment in first-time mothers. Front Psychol. 2015;6:1443.

73. McFarland J, Salisbury AL, Battle CL, Hawes K, Halloran K, Lester BM. Major depressive disorder during pregnancy and emotional attachment to the fetus. Arch Womens Ment Health. 2011;14:425–34.

74. Mehran P, Simbar M, Shams J, Ramezani-Tehrani F, Nasiri N. History of perinatal loss and maternal–fetal attachment behaviors. Women and Birth. 2013;26:185–89.

75. Mercer RT, Ferkehch SL. Predictors of parental attachment during early parenthood. J Adv Nurs. 1990;15:268–80.

76. Mercer RT, Ferketich SL. Maternal-infant attachment of experienced and inexperienced mothers during infancy. Nurs Res. 1994;43:344–51.

77. Mikulincer M, Florian V. Maternal-fetal bonding, coping strategies, and mental health during pregnancy–the contribution of attachment style. J Soc Clin Psychol. 1999;18:255–76.

78. Moehler E, Brunner R, Wiebel A, Reck C, Resch F. Maternal depressive symptoms in the postnatal period are associated with long-term impairment of mother–child bonding. Arch Womens Ment Health. 2006;9:273–78.

79. Muller ME. The development and testing of the Mueller Prenatal Attachment Inventory. 1990.

80. Müller ME. A questionnaire to measure mother-to-infant attachment. J Nurs Meas. 1994;2:129–41.

81. Muzik M, Bocknek EL, Broderick A, et al. Mother–infant bonding impairment across the first 6 months postpartum: the primacy of psychopathology in women with childhood abuse and neglect histories. Arch Womens Ment Health. 2013;16:29–38.

82. Nolvi S, Karlsson L, Bridgett DJ, Pajulo M, Tolvanen M, Karlsson H. Maternal postnatal psychiatric symptoms and

infant temperament affect early mother-infant bonding. Infant Behavior & Development. 2016;43:13–23.

83. Nonnenmacher N, Noe D, Ehrenthal JC, Reck C. Postpartum bonding: the impact of maternal depression and adult attachment style. Arch Womens Ment Health. 2016;19:927–35.

84. O’Higgins M, Roberts ISJ, Glover V, Taylor A. Mother-child bonding at 1 year; associations with symptoms of postnatal depression and bonding in the first few weeks. Arch Womens Ment Health. 2013;16:381–89.

85. Ohoka H, Koide T, Goto S, et al. Effects of maternal depressive symptomatology during pregnancy and the postpartum period on infant–mother attachment. Psychiatry Clin Neurosci. 2014;68:631–39.

86. Örün E, Yalçın SS, Mutlu B. Relations of maternal psychopathologies, social-obstetrical factors and mother-infant bonding at 2-month postpartum: a sample of Turkish mothers. World J Pediatr. 2013;9:350–55.

87. Ossa X, Bustos L, Fernandez L. Prenatal attachment and associated factors during the third trimester of pregnancy in Temuco, Chile. Midwifery. 2012;28:e689–e696.

88. Pascoe JM, Kokotailo PK, Broekhuizen FF. Correlates of multigravida women’s binge drinking during pregnancy: a longitudinal study. Arch Pediatr Adolesc Med. 1995;149:1325–29.

89. Pearson RM, Lightman SL, Evans J. Attentional processing of infant emotion during late pregnancy and mother–infant relations after birth. Arch Womens Ment Health. 2011;14:23–31.

90. Petri E, Palagini L, Bacci O, et al. Maternal–foetal attachment independently predicts the quality of maternal–infant bonding and post-partum psychopathology. J Matern Neonatal Med. 2017:1–7.

91. Pires de Almeida C, Sá E, Cunha F, Pires EP. Violence during pregnancy and its effects on mother–baby relationship during pregnancy. J Reprod Infant Psychol. 2013;31:370–80.

92. Pollmann MTF, Hoffenaar PJ. Haptonomische zwangerschapsbegeleiding en de prenatale gehechtheid van ouders aan hun kindHaptonomic guidance of pregnancy and the prenatal attachment of both parents to their unborn child. Kind en Adolesc. 2017;38:108–19.

93. Reck C, Klier CM, Pabst K, et al. The German version of the Postpartum Bonding Instrument: psychometric properties and association with postpartum depression. Arch Womens Ment Health. 2006;9:265–71.

94. Reck C, Zietlow A-L, Müller M, Dubber S. Perceived parenting stress in the course of postpartum depression: the buffering effect of maternal bonding. Arch Womens Ment Health. 2016;19:473–82.

95. Ricbourg A, Gosme C, Gayat E, Ventre C, Barranger E, Mebazaa A. Emotional impact of severe post-partum haemorrhage on women and their partners: an observational, case-matched, prospective, single-centre pilot study. Eur J Obstet Gynecol Reprod Biol. 2015;193:140–43.

96. Rossen L, Hutchinson D, Wilson J, et al. Predictors of postnatal mother-infant bonding: the role of antenatal bonding, maternal substance use and mental health. Arch Womens Ment Health. 2016;19:609–22.

97. Rowe H, Fisher J, Quinlivan J. Women who are well informed about prenatal genetic screening delay emotional attachment to their fetus. J Psychosom Obstet Gynecol. 2009;30:34–41. doi:10.1080/01674820802292130.

98. Rowe HJ, Wynter KH, Steele A, Fisher JRW, Quinlivan JA. The growth of maternal-fetal emotional attachment in pregnant adolescents: a prospective cohort study. J Pediatr Adolesc Gynecol. 2013;26:327–33.

99. Schodt CM. Patterns of parent-fetus attachment and the couvade syndrome: An application of human-environment integrality as postulated in the science of unitary human beings. 1989.

100. Schwerdtfeger KL, Goff BSN. Intergenerational transmission of trauma: Exploring mother–infant prenatal attachment. J Trauma Stress. 2007;20:39–51.

101. Scopesi A, Viterbori P, Sponza S, Zucchinetti P. Assessing mother‐to‐infant attachment: the Italian adaptation of a self‐report questionnaire. J Reprod Infant Psychol. 2004;22:99–109.

102. Seimyr L, Sjögren B, Welles-Nyström B, Nissen E. Antenatal maternal depressive mood and parental–fetal attachment at the end of pregnancy. Arch Womens Ment Health. 2009;12:269–79.

103. Şen S, Kavlak O. Transgenerational attachment in Manisa, Turkey. Contemp Nurse. 2012;41:126–32.

104. Shin H, Kim YH. Maternal Attachment Inventory: psychometric evaluation of the Korean version. J Adv Nurs. 2007;59:299–307.

105. Sjögren B, Edman G, Widström AM, Mathiesen AS, Uvnäs‐Moberg K. Maternal foetal attachment and personality during first pregnancy. J Reprod Infant Psychol. 2004;22:57–69.

106. Stanton F, Golombok S. Maternal-fetal attachment during pregnancy following in vitro fertilization. J Psychosom Obstet Gynaecol. 1993;14:153–58.

107. St. John BLW. The Relationships Among Prenatal Attachment, Social Support, Prenatal Care Utilization and the Nutritional Status of Pregnant Women. 2002.

108. Suetsugu Y, Honjo S, Ikeda M, Kamibeppu K. The Japanese version of the Postpartum Bonding Questionnaire: Examination of the reliability, validity, and scale structure. J Psychosom Res. 2015;79:55–61.

109. Tani F, Castagna V, Ponti L. Women who had positive relationships with their own mothers reported good attachments to their first child before and after birth. Acta Paediatr. 2018;107:633–37.

110. Taylor A, Atkins R, Kumar R, Adams D, Glover V. A new Mother-to-Infant Bonding Scale: links with early maternal mood. Arch Women’s Ment Heal. 2005;8:45–51.

111. Teixeira M, Raimundo F, Antunes M. Relation between Maternal-Fetal Attachment and Gestational Age and Parental Memories. Journal of Nursing Referencia. 2016:85–92.

112. Tikotzky L. Postpartum Maternal Sleep, Maternal Depressive Symptoms and Self-Perceived Mother–Infant Emotional Relationship. Behav Sleep Med. 2016;14:5–22.

113. Tsartsara E, Johnson MP. The impact of miscarriage on women’s pregnancy-specific anxiety and feelings of prenatal maternal–fetal attachment during the course of a subsequent pregnancy: An exploratory follow-up study. J Psychosom Obstet Gynecol. 2006;27:173–82.

114. Ustunsoz A, Guvenc G, Akyuz A, Oflaz F. Comparison of maternal-and paternal-fetal attachment in Turkish couples. Midwifery. 2010;26:e1–e9.

115. Wachter M. Psychological distress and dyadic satisfaction as predictors of maternal-fetal attachment. 2003.

116. Walsh J, Hepper EG, Bagge SR, Wadephul F, Jomeen J. Maternal–fetal relationships and psychological health: emerging research directions. J Reprod Infant Psychol. 2013;31:490–99.

117. Wang L, Mills A. The Relationship of Maternal-Fetal Attachment and Health Behavior Among Pregnant Women in South Taiwan. 2012.

118. Weatherby F. Role expectation discrepancy: A comparison of nonobese, moderately obese and excessively obese primigravidas. 1989.

119. Wilkinson RB, Scherl FB. Psychological health, maternal attachment and attachment style in breast‐ and formula‐feeding mothers: a preliminary study. J Reprod Infant Psychol. 2006;24:5–19.

120. Wittkowski A, Wieck A, Mann S. An evaluation of two bonding questionnaires: a comparison of the Mother-to-Infant Bonding Scale with the Postpartum Bonding Questionnaire in a sample of primiparous mothers. Arch Womens Ment Health. 2007;10:171–75.

121. Wu JHL, Eichmann MA. Fetal Sex Identification and Prenatal Bonding. Psychol Rep. 1988;63:199–202.

122. Yalçın SS, Örün E, Mutlu B, et al. Why are they having infant colic? A nested case-control study. Paediatr Perinat Epidemiol. 2010;24:584–96.

123. Zachariah R. Maternal–fetal attachment: Influence of mother–daughter and husband–wife relationships. Res Nurs Health. 1994;17:37–44.

124. Zanardo V, Gabrieli C, Straface G, Savio F, Soldera G. The interaction of personality profile and lactation differs between mothers of late preterm and term neonates. J Matern Neonatal Med. 2017;30:927–32.

125. Zanardo V, Bertin M, Sansone L, Felice, L. The adaptive psychological changes of elective induction of labor in breastfeeding women. Early Hum Dev. 2017;104:13–16.

126. Zanardo V, Soldera G, Volpe F, Giliberti L, Parotto M, Giustardi A, Straface G. Influence of elective and emergency cesarean delivery on mother emotions and bonding. Early Hum Dev. 2016;99:17–20.

127. Zdolska-Wawrzkiewicz A, Chrzan-Dętkoś M, Bidzan M. Maternal attachment style during pregnancy and becoming a mother in Poland. J Reprod Infant Psychol. 2018;36:4–14.

128. Zeitlin D, Dhanjal T, Colmsee M. Maternal-foetal bonding: the impact of domestic violence on the bonding process between a mother and child. Arch Womens Ment Health. 1999;2:183–89.

129. Zhang H, Su Q, Yao D, et al. Prolactin, a potential mediator of reduced social interactive behavior in newborn infants following maternal perinatal depressive symptoms. J Affect Disord. 2017;215:274–80.

130. Zimerman A, Doan H, McK. Prenatal attachment and other feelings and thoughts during pregnancy in three groups of pregnant women. J Prenatal Perinatal Psychol Health. 2003;18:131–48.
