# Supplementary material for: Correlates of prenatal and postnatal mother-to-infant bonding quality: A systematic review
Source: PLoS One. 2019 Sep 24;14(9):e0222998. doi: 10.1371/journal.pone.0222998 (PMC6759162; doi:10.1371/journal.pone.0222998)
Supplement: S1 Table — (DOCX) [file pone.0222998.s005.docx]

**S1 Table** List of in- and excluded measurements according to the definition of mother-to-infant bonding; the emotional or affective tie (feelings) experienced by a mother towards her child.

| **Included outcome measurements in final search** | **Definition** |
| --- | --- |
| Antenatal Maternal Attachment Scale: AMAS (Honjo et al., 2003) | The **bond of love** formed between one person and another particular person (in this case, fetus). |
| Maternal Antenatal Attachment Scale: MAASAntenatal Bonding Questionnaire (Condon, 1993) | Contains items focusing upon **feelings, behaviours and attitudes towards the fetus.** |
| Maternal Attachment Inventory: MAI (Muller, 1994) also known as Muller's Maternal Attachment Inventory | The Maternal Attachment Inventory (MAI) was developed and tested to provide a practical measure of **maternal affectionate attachment**. |
| Maternal Fetal Attachment Scale: MFAS (Cranley 1981) | self-report questionnaire to measure the **antenatal maternal feeling towards the unborn baby**, six aspects of the early bonding of the expectant woman to the fetus |
| Maternal Postnatal Attachment Scale: MPAS (Condon & Corkindale, 1998)  Maternal Postnatal Attachment Questionnaire (MPAQ) | A self-report questionnaire designed to assess a **mother’s subjective feelings of attachment to her infant**. |
| Modified Maternal Foetal Attachment Scale: MMFAS (Hsu and Chen, 2001) | Hsu and Chen (2001, quoted in Hang et al, 2004) **merged the items of the MFAS with those of the PAI** and developed the Modified Maternal Foetal Attachment Scale; |
| Mother-to-infant bonding scale: MIB (Taylor et al. 2005)  Also known as Mother-Infant Bonding Scale (MIBS), Mother-Baby Bonding Scale  Mother-to-infant bonding measure  Mother-Infant Bonding Questionnaire (MIBQ) | The items of this questionnaire are a list of adjectives describing **feelings mothers have towards their baby**.  MIB consists statements describing an emotional response (loving, resentful, neutral or felt nothing, joyful, dislike, protective, disappointed, aggressive) |
| Postpartum Bonding Questionnaire: PBQ (Brockington, Fraser & Wilson, 2006)  Shortened-Postpartum Bonding Questionnaire: S-PBQ SPBQ | The Postpartum Bonding Questionnaire, PBQ, measures the **quality of the parent-baby bond**. |
| Pregnancy involvement list: PIL (Kleinveld et al., 2007) | **Attachment very early in pregnancy**  ‘I look forward to the birth of my child’, ‘I try to imagine what my child will look like’, ‘I look after my health more now that I’m pregnant’, ‘I’m looking for things for the baby’, ‘I think about how I will bring up my child’. |
| Prenatal Attachment Inventory: PAI (Muller, 1993) Prenatal Atachment Inventory-Revised: PAI-R | ‘the unique, **affectionate relationship that develops between a woman and her fetus**’. The goal was to provide an additional tool, which focused on affiliation, rather than on the behaviours |
| the How I Feel About the Baby Now Scale: FAB or HIFBN (Leiffer 1977) | **Feelings about the baby.**  I feel tenderly towards my baby, I feel annoyed at my baby, I feel curious about my baby |
| Parent-to-infant Attachment Questionnaire: PAQ | **Feelings about the baby.** |
| Pre- and Postnatal Bonding Scale (PPBS) | **Feelings about the baby prenatal and postnatal** |

**NOTE**: Mother-to-infant bonding instruments are included which, in accordance with the definition of mother-to-infant bonding, focus on the emotions and feelings experienced by a mother towards her child. So, we included instruments with a total mother-to-infant bonding score, which means that if only subscales of mother-to-infant bonding were reported as for example rejection and anger the reported subscales do not reflect the construct of mother-to-infant bonding as a whole.

| **Excluded outcome measurements in final search** | **This review excluded studies which use a** |
| --- | --- |
| Strange situation test  Attachment Q-set Attachment Q-Sort (AQS)  Attachment to parent scale  CARE-index procedure = CARE-index  Child attachment interview  Global Ratings for Mothers-Infant Interactions/ Global Ratings Scales  Manchester Child Attachment Story Task (MCAST)  Marschak Interaction Method Behavioral Rating System (MIMBRS)  Parent Child Early Relational Assessment (PCERA)  Parent-Child Interaction Questionnaire (PCIQ)  Preschool Assessment of Attachment (PAA)  School-aged Assessment of Attachment (SAA) | (1) measurement of attachment or bonding from the **perspective of the child** or an i**nteraction** because this does not measures the relationship between a mother and her child from only the perspective of the mother |
| Family Relationship Questionnaire (FRQ)  Emotional Availability Scales  child parent relationship scale (CPRS )  Avant Maternal Attachment Behavior Scale  Attachment Style Questionnaire (ASQ)(Feeney et al.,1994)  Hechtingsstijllijst (HSL)  Interpersonal mindfulness in parenting scale  IPARTheory (formerly known as parental acceptance)  Maternal Adjustment and Maternal Attitudes (MAMA)  Maternal attitude questionnaire  Maternal Self-Efficacy Scale  maternal sensitivity (scales) Ainsworth's Maternal Sensitivity Scale (AMSS) Coding Interactive Behavior (CIB) Emotional Availability Scales  Global Ratings of Mother Infant Interaction  Maternal Behaviour Q-sort (MBQS)  Pederson and Moran Sensitivity Q-Sort  maternity experiences scale  Mother and Baby Interaction Scale (MABIS)  Mothers’ Object Relations Scale – Short Form (MORS-SF)  Neonatal perception inventory (NPI)  Parent-Infant Relationship Global Assessment Scale (PIR-GAS)  Parental Meta emotions (interview)  parenting Alliance Measure (PAM)  Pictorial Representation of Attachment Measure: PRAM  prenatal parental reflective functioning (P-PRF)  Toddler Attachment Scale Sort -39/ Sort -45  Yale Inventory of Parental Thoughts and Actions (YIPTA) | (2) measurement where **the feelings** of the mother towards her child **are not the primary indicator** of the measurement. They measure other concepts like behavior, perceptions, parental sensitivity (mother's ability to perceive and infer the meaning behind her infant's [behavioural signals](https://en.wikipedia.org/wiki/Human_behavior), and to respond to them promptly and appropriately) parental representations, mindful parenting or interaction |
| Adult attachment interview (AAI) Adult Attachment Projective (AAP) Adult Attachment Style Scale (AASS) Adult Separation Anxiety (ASA) Adult separation anxiety disorder (ASAD) Intimate Bonds Measure IBM  Revised Adult Attachment Scale (RAAS) | (3) measurement of **adult attachment in genera**l and not specific from mother to child. |
| Adult scale of parental attachment (ASPA) Parental Bonding Instrument (PBI) | (1) measurement of attachment or bonding from the **perspective of the child** and  (3) measurement of **adult attachment in general** and not specific from mother to child. |
| Birmingham Interview for Maternal Mental Health: BIMMH (Chandra, 2006) parental feelings inventory within the parenting role  Parent child relationship inventory (PCRI) | (2) where **the feelings** of the mother towards her child **are not the primary indicator** of the measurement, only a small part of the interview consist themes of feelings |
| Clinical Interview for Parents of High-risk Infants (CLIP) | (4) measurement is not for the general population, is meant for a subgroup. |
