# Supplementary material for: Correlates of prenatal and postnatal mother-to-infant bonding quality: A systematic review
Source: PLoS One. 2019 Sep 24;14(9):e0222998. doi: 10.1371/journal.pone.0222998 (PMC6759162; doi:10.1371/journal.pone.0222998)
Supplement: S2 Table — (DOCX) [file pone.0222998.s006.docx]

**S2 Table** Overview of included studies examining associations between demographic, reproduction-related, psychosocial,

child-related and partner-related correlates and mother-to-infant bonding quality (prenatal and postnatal).

| **nr** | **First author** | **Year** | **Country** | **Setting^a^** | **Design** | **Analyses** |  | **Sample demographics** | | | **Instruments** |
| --- | --- | --- | --- | --- | --- | --- | --- | --- | --- | --- | --- |
|  |  |  |  |  | **of the analyses^c^** | **uni- or multi- variable** | **n in analyses Range** | **Age**  **Mean (sd)** | **Nulliparae^d^**  **%** | **Caucasian**  **%** |  |
| 1 | Abuhammad, S [1] | 2016 | Jordan | Care | CS | uni+multi | 258 | 27.5 (5.9) | NR | NR | MAI |
| 2 | Alan, H [2] | 2013 | Turkey | Care | CS | uni+multi | 135 | 26.6 (5.5) | NR | NR | MAI |
| 3 | Andrek, A [3] | 2016 | Hungary | Care | CS | uni | 107 – 114 | 31.8 (4.3) | 70 | NR | MFAS |
| 4 | Armstrong, D [4] | 1998 | USA | Open | CS | uni | 15 - 30 | 29 | NR | NR | PAI |
| **5** | **Barone, L** [5] | **2014** | **Italy** | **Care** | **CS** | **multi** | **130** | **33.9 (5.7)** | **62** | **NR** | **PAI** |
| 6 | Bennington, L[6] | 2012 | USA | Open | CS | uni+multi | 273 - 314 | 30.7 | 50 | 86 | MAI |
| 7 | Berryman, J [7] | 1996 | UK | Care | CS | uni | 96 | NR | 49 | NR | MFAS |
| **8** | **Bicking Kinsey, C** [8] | **2014** | **USA** | **Open** | **CS + PC** | **uni+multi** | **2798** | **27.6 (4.3)** | **100** | **86** | **S-PBQ10** |
| 9 | Bielawska-Batorowicz, E [9] | 2008 | Sweden + Poland | Care | CS | multi | 171 – 409 | 29.1 | 47 | 100 | PAI |
| 10 | Boztepe, H [10] | 2016 | Turkey | Care ^e^ | CS | uni | 200 | NR | NR | NR | MAI |
| 11 | Busonera, A [11] | 2016 | Italy | Care | CS + PC | uni | 100 – 482 | NR | 85 | 100 | MFAS+MPAS |
| 12 | Bussel, van J [12] | 2010 | Belgium | Care | CS + PC | uni | 198 – 258 | 30.4 (4.1) | 48 | NR | PBQ+MIBS+  MPAS |
| 13 | Calhoun, A [13] | 2015 | USA | Care | PC | uni+multi | 107 | 27.9 (6.1) | 26 | NR | MIBS |
| 14 | Cetisli, N [14] | 2018 | Turkey | Care ^e^ | CS | uni | 175 | 24.3 | 100 | NR | MAI |
| 15 | Chang, H [15] | 2016 | Taiwan | Care | CS | uni | 300 | 31.6 (4.1) | 65 | NR | MMFAS |
| 16 | Chazotte, C [16] | 1995 | USA | Care | CS | uni | 60 | 25.3 | 38 | 7 | MFAS |
| **17** | **Chen, C** [17] | **2011** | **Taiwan** | **Care** | **CS + PC** | **uni** | **110 – 125** | **NR** | **55** | **NR** | **MFAS+MAI** |
| 18 | Cinar, N [18] | 2015 | Turkey | Care | CS | uni | 122 | 25.0 (2.2) | NR | NR | MAI |
| **19** | **Cock de, E** [19] | **2017** | **Netherlands** | **Care** | **CS + PC** | **uni** | **335** | **31.7(4.2)** | **55** | **NR** | **MAAS+MPAS** |
| 20 | Condon, J [20] | 1997 | Australia | Care | CS | uni+multi | 236 | 27.4 (4.7) | 33 | NR | MAAS |
| 21 | Cranley, M [21] | 1981 | USA | Care | CS | uni | 30 | 27 | 27 | 97 | MFAS |
| 22 | Cranley, M [22] | 1984 | USA | Care + open | CS | uni | 326 | NR | 56 | NR | MFAS |
| 23 | Delavari, M [23] | 2018 | Iran | Care | CS | uni+multi | 287 | NR | 100 | NR | MFAS |
| 24 | Della Vedova, A [24] | 2008 | Italy | Open | CS | uni | 184 – 214 | NR | 81 | NR | PAI |
| 25 | Della Vedova, A [25] | 2011 | Italy | Care + open | CS | uni | 146 | 31.3 (3.8) | 100 | NR | PAI |
| 26 | Denis, A [26] | 2015 | France | Care + Open | CS | uni | 117 | 27.5 (4.3) | 100 | NR | MAAS |
| 27 | Doster, A [27] | 2018 | Germany | Care | CS + PC | uni | 152 - 324 | 32.8 (4.6) | 23 | NR | MFAS+PBQ16 |
| **28** | **Dubber, S** [28] | **2015** | **Germany** | **Care** | **CS + PC** | **uni+multi** | **30 – 79** | **32.8 (4.4)** | **NR** | **NR** | **PBQ16** |
| **29** | **Edhborg, M** [29] | **2005** | **Sweden** | **Care** | **CS + PC** | **multi** | **106** | **32.1 (4.4)** | **52** | **NR** | **PBQ** |
| **30** | **Edhborg, M** [30] | **2011** | **Bangladesh** | **Care** | **CS + PC** | **uni+multi** | **663 – 672** | **24.6 (6.1)** | **27** | **NR** | **PBQ** |
| 31 | Feldstein, S [31] | 2004 | USA | open | CS | uni | 59 | 33.6 (4.7) | NR | NR | MPAQ |
| 32 | Figueiredo, B [32] | 2007 | Portugal | Care | CS | uni+multi | 59 - 72 | NR | 100 | 99 | New MIBS |
| 33 | Figueiredo, B [33] | 2009 | Portugal | Care | CS | uni+multi | 114 - 312 | 26.6 | 68 | 99 | PBS |
| 34 | Foster, S [34] | 1996 | UK | Care | CS | uni | 38 | 29 (5.2) | NR | NR | MFAS |
| 35 | Friedman, K [35] | 2008 | USA | Care + open | CS | uni | 50 | 32.4 (4.8) | NR | 88 | PBQ |
| 36 | Fuller, S [36] | 1993 | USA | Primary | CS | uni+multi | 337 | 23.7 (4.3) | NR | 51 | MFAS |
| 37 | Gaffney, K [37] | 1986 | USA | Open | CS | uni | 100 | 26 | 67 | NR | MFAS |
| 38 | Garcia, L [38] | 2016 | Spain | Care | CS | uni | 513 | 34 | NR | NR | PBQ |
| **39** | **Gau, M** [39] | **1996** | **USA** | **Care** | **CS** | **uni** | **349** | **29.2 (5.3)** | **78** | **71** | **PAI** |
| 40 | Gaudet, C [40] | 2010 | France | Care + open | CS | uni | 170 | 28.6 | 59 | NR | MAAS |
| **41** | **Gharaibeh, M** [41] | **2012** | **Jordan** | **Care** | **CS** | **uni+multi** | **220** | **24.3 (4.7)** | **100** | **NR** | **MAI** |
| 42 | Goecke, T [42] | 2012 | Germany | Care | CS | uni | 161 | 29.9 (4.0) | 100 | NR | MAAS |
| 43 | Grace, J [43] | 1989 | USA | Care | CS + PC | uni | 69 | 27.5 (3.8) | 42 | NR | MFAS |
| 44 | Haedt, A [44] | 2007 | USA | Care + open | CS | uni+multi | 188 | 28.8 (5.4) | 72 | 88 | MFAS |
| **45** | **Hairston, I** [45] | **2011** | **USA** | **Open** | **PC** | **uni** | **184** | **27.45** | **NR** | **60** | **PBQ** |
| **46** | **Hall, R** [46] | **2015** | **Netherlands** | **Care** ^e^ | **CS + PC** | **multi** | **211** | **31.8** | **65** | **NR** | **PBQ** |
| 47 | Handelzalts, J [47] | 2016 | Israel | Care ^e^ | CS + PC | uni+multi | 58 – 89 | 30.2 | NR | NR | MFAS+MIBS |
| 48 | Harpel, T [48] | 2018 | USA | Open | CS | uni | 117 | NR | 50 | 83 | MAAS |
| 49 | Hergüner, S [49] | 2014 | Turkey | Care | CS | uni | 80 | 28.8 | NR | NR | MAI |
| 50 | Himani [50] | 2011 | India | Care | PC | uni | 218 | 26.4 | 39 | NR | MPAS |
| **51** | **Hjelmstedt, A^b^** [51] | **2006** | **Sweden** | **Care** ^e^ | **PC** | **uni** | **90** | **31.9** | **100** | **NR** | **PAI** |
| 52 | Høivik, M [52] | 2013 | Norway | Primary | CS + PC | uni | 20 – 52 | 29.4 | NR | 100 | PBQ |
| 53 | Honjo, S [53] | 2003 | Japan | Care | CS | uni | 214 – 216 | 30.5 (4.4) | NR | NR | AMAS |
| 54 | Horsch, A [54] | 2017 | Switzerland | Open ^e^ | CS | uni | 186 | 32.3 | 35 | NR | MIBS |
| 55 | Hsu, T [55] | 2001 | Taiwan | Care | CS | uni+multi | 150 | 29.5 | 49 | NR | MMFAS |
| 56 | Kaminer, A [56] | 1992 | USA | Care ^e^ | CS | uni | 60 – 132 | 25.2 | 35 | 32 | MFAS |
| 57 | Kaneko, H [57] | 2014 | Japan | Care | CS | uni | 1786 | 29.8 (5.0) | 45 | NR | PBQ |
| 58 | Kemp, V [58] | 1987 | USA | Secondary+ open | CS | uni | 85 | 28.2 | 72 | 75 | MFAS |
| **59** | **Kita, S** [59] | **2016** | **Japan** | **Care** | **CS + PC** | **uni** | **562** | **32.2 (4.9)** | **52** | **NR** | **MIBS** |
| **60** | **Kokubu, M** [60] | **2012** | **Japan** | **Care** | **CS + PC** | **uni** | **99** | **29.9 (4.1)** | **57** | **NR** | **MIBS** |
| 61 | Kraft, A [61] | 2017 | Germany | Care | PC | uni | 46 | NR | NR | NR | MAAS+MPAS |
| 62 | Krisjanous, J [62] | 2014 | New Zealand | Care | CS | uni | 478 | NR | 100 | 75 | PAI |
| 63 | Kunkel [63] | 2003 | Canada | Open | CS | uni | 35 | NR | 69 | NR | MFAS |
| 64 | Lahann, R [64] | 2008 | USA | Open | CS | uni | 168 | 30.5 (5.1) | 54 | 91 | PAI |
| 65 | Lear, T [65] | 2013 | USA | Care | CS | uni | 177 – 201 | 32.3 (4.4) | 51 | 67 | PAI |
| 66 | Lee, T [66] | 1994 | Taiwan | Care | CS + PC | uni | 77 | 29.6 | 100 | NR | MFAS |
| 67 | Lerum, C [67] | 1989 | USA | Care | CS | uni | 72 – 80 | 24.9 (4.2) | NR | NR | MFAS |
| 68 | Levine, A [68] | 2007 | Israel | Care | CS + PC | uni | 45 - 62 | 28.2 | 49 | NR | MFAS |
| 69 | Lindgren, K [69] | 2001 | USA | Care | CS | uni+multi | 252 | 29.5 (6.1) | 41 | 77 | MFAS |
| 70 | Lingeswaran, A [70] | 2012 | India | Care | CS | uni | 230 | 22.9 (2.7) | 76 | NR | MFAS |
| 71 | Mason, Z [71] | 2009 | USA | Care | CS | uni | 284 | 23.5 | 100 | 3 | MPAS |
| 72 | Mazzeschi, C [72] | 2015 | Italy | Care | CS | uni | 70 | 32.8 (4.8) | 100 | NR | MAAS |
| 73 | McFarland, J [73] | 2011 | USA | Care ^e^ | PC | uni | 123 | 29.1 | 30 | 70 | MFAS |
| 74 | Mehran, P [74] | 2013 | Iran | Care | CS | uni | 100 | 26.1 | NR | NR | MFAS |
| 75 | Mercer, R [75] | 1990 | USA | Secondary + primary | PC | uni | 194 – 281 | 28.7 | 55 | 71 | HIFABNS |
| 76 | Mercer, R [76] | 1994 | USA | Care | CS + PC | uni | 182 – 302 | 28.5 | 55 | 71 | FAB+MFAS |
| 77 | Mikulincer, M [77] | 1999 | Israel | Care | CS | uni | 260 | 28 (3.6) | 100 | NR | MFAS |
| 78 | Mikulincer, M [77] | 1999 | Israel | Care | PC | uni | 30 | 27 (2.9) | 100 | NR | MFAS |
| 79 | Moehler, E [78] | 2006 | Germany | Care | CS + PC | uni+multi | 101 | 33.3 | NR | 100 | PBQ |
| 80 | Müller, M [79] | 1990 | USA | Care + open | CS | uni | 292 – 310 | 30 | 63 | 68 | PAI+MFAS |
| 81 | Müller, M [80] | 1994 | USA | Open | PC | uni | 62 – 84 | 27.9 | NR | 91 | MAI |
| 82 | Muzik, M [81] | 2013 | USA | Care + open | PC | uni | 150 | 29 (5.7) | NR | 67 | PBQ |
| 83 | Nolvi, S[82] | 2016 | Finland | Care | CS + PC | uni+multi | 62 – 102 | 29.9 (4.9) | 62.5 | NR | PBQ |
| 84 | Nonnenmacher, N [83] | 2016 | Germany | Care ^e^ | PC | uni | 93 | 34.0 (4.5) | NR | NR | PBQ16 |
| 85 | O'Higgins, M [84] | 2013 | UK | Care | CS + PC | uni | 79 | 33.1 | 76 | 76 | MIBS |
| 86 | Ohoka, H [85] | 2014 | Japan | Care | CS + PC | uni | 389 | 31.7 (4.3) | 65 | NR | MIBS |
| 87 | Örün, E [86] | 2013 | Turkey | Care | CS + PC | uni | 189 | 25.1 (5.2) | 43 | NR | PBQ |
| 88 | Ossa, X [87] | 2012 | Chile | Care | CS | uni+multi | 243 | 25 (6) | 44 | NR | MAAS |
| 89 | Pascoe, J [88] | 1995 | USA | Care | CS | uni | 101 – 139 | 24.5 | 24 | NR | MFAS |
| 90 | Pearson, R [89] | 2011 | UK | Care | CS + PC | uni+multi | 49 | 29.5 | 50 | 95 | PBQ |
| 91 | Petri, E [90] | 2017 | Italy | Care | PC | uni+multi | 106 | 33.9 (4.0) | 72 | NR | MPAS |
| 92 | Pires de Almeida, C [91] | 2013 | Portugal | Care | CS | uni | 184 | NR | NR | NR | MFAS |
| 93 | Pollmann, M [92] | 2017 | Netherlands | Care ^e^ | CS + PC | uni | 38 – 40 | NR | 63 | NR | MAAS+MFAS |
| 94 | Reck, C [93] | 2006 | Germany | Care | CS | uni | 862 | 33 (4.6) | NR | NR | PBQ+PBQ16 |
| 95 | Reck, C [94] | 2016 | Germany | Care ^e^ | CS + PC | uni | 35 – 50 | 32.3 (4.7) | 64 | NR | PBQ16 |
| 96 | Ricbourg, A [95] | 2015 | France | Care ^e^ | PC | uni | 34 – 40 | 30.8 | NR | NR | MIBS |
| **97** | **Rossen, L**[96] | **2016** | **Australia** | **Care** ^e^ | **CS + PC** | **uni+multi** | **367 – 372** | **32.6 (5.0)** | **40** | **NR** | **MAAS+MPAS** |
| **98** | **Rowe, H** [97] | **2009** | **Australia** | **Secondary** | **CS + PC** | **uni+multi** | **64 – 134** | **29.1 (4.7)** | **50** | **NR** | **MAAS** |
| **99** | **Rowe, H** [98] | **2013** | **Australia** | **Care** ^e^ | **PC** | **uni+multi** | **200 – 299** | **22.9** | **52** | **NR** | **MAAS** |
| 100 | Schodt, C [99] | 1989 | USA | Care | CS | uni | 110 | 30.7 (4.3) | 84 | NR | MFAS |
| 101 | Schwerdtfeger,K[100] | 2007 | USA | Care | CS | uni+multi | 41 | 22.9 (3.8) | 46 | 54 | MAAS |
| 102 | Scopesi, A [101] | 2004 | Italy | Care | CS | uni | 195 – 210 | 32.1 (4.4) | 62 | NR | MPAS |
| **103** | **Seimyr, L** [102] | **2009** | **Sweden** | **Care** | **CS** | **uni+multi** | **298** | **29 (4.7)** | **NR** | **NR** | **MFAS** |
| 104 | Sen, S [103] | 2012 | Turkey | Care | CS | uni | 140 | 26.9 (5.2) | NR | NR | MAI |
| 105 | Shin, H [104] | 2007 | South-Korea | Care | CS | uni | 196 | 31 (3.9) | NR | NR | MAI |
| 106 | Sjögren, B [105] | 2004 | Sweden | Care | CS | uni | 76 | 26.8 | 100 | NR | MFAS |
| 107 | Stanton, F [106] | 1993 | UK | Care | CS | uni | 35 | 31.7 | 91 | NR | MFAS |
| 108 | St John, B [107] | 2002 | USA | Open | CS | uni+multi | 142 | NR | NR | NR | PAI |
| 109 | Suetsugu, Y [108] | 2015 | Japan | Care | CS + PC | uni | 199 – 244 | 30.6 (4.7) | NR | NR | PBQ+PBQ14 |
| 110 | Tani, F [109] | 2018 | Italy | Care | CS | uni | 201 | 32.0 (4.8) | 100 | NR | PAI |
| **111** | **Taylor, A** [110] | **2005** | **UK** | **Care** | **CS + PC** | **uni+multi** | **144 – 162** | **31.9 (4.6)** | **69** | **NR** | **New MIBS** |
| 112 | Teixeira, M [111] | 2016 | Portugal | Care | CS | uni | 179 | NR | NR | NR | MFAS |
| 113 | Tikotzky, L [112] | 2016 | Israel | Open | CS | uni+multi | 78 | 30.8 (4.8) | 57 | NR | PBQ+MPAS |
| 114 | Tsartsara, E [113] | 2006 | UK | Care | CS + PC | uni+multi | 24-35 | 30.4 (6.1) | 57 | NR | MAAS |
| 115 | Ustunsoz, A [114] | 2010 | Turkey | Care | CS | uni+multi | 144 | 26.7 (5.3) | NR | NR | PBQ |
| 116 | Wachter, M [115] | 2003 | USA | Care | CS | uni+multi | 62 | 28.5 (4) | 100 | 44 | MFAS |
| 117 | Walsh, J [116] | 2013 | UK | Open | CS | uni | 258 | 31.8 (4.5) | 48 | 89 | MFAS |
| 118 | Wang, L[117] | 2012 | Taiwan | Care | CS | uni | 390 | 29.5 (4.5) | 54 | NR | MFAS |
| 119 | Weatherby, F [118] | 1989 | USA | Care | CS | uni | 97 | 20.5 | 100 | 67 | MFAS |
| 120 | Wilkonson, R [119] | 2006 | Australia | Care + open | CS | uni | 60 | 30.2 | 60 | 86 | MAI |
| 121 | Wittkowski, A [120] | 2007 | UK | Care | CS + PC | uni | 96 | 28.8 (5.1) | 100 | NR | PBQ+MIBS |
| 122 | Wu, J [121] | 1988 | USA | Care | CS | uni | 57 | NR | NR | 80 | MFAS |
| 123 | Yalçin, S [122] | 2010 | Turkey | Care | CS | uni | 189 | 25.2 | NR | NR | MIBS |
| 124 | Zachariah, R [123] | 1994 | USA | Open | CS | uni | 115 | 24.9 (3.0) | 100 | 100 | MFAS |
| 125 | Zanardo, V [124] | 2017 | Italy | Care | PC | uni | 90 | 35.2 | 52 | NR | MIBS |
| 126 | Zanardo, V [125] | 2017 | Italy | Care | PC | uni | 180 | NR | 58 | NR | MIBS |
| **127** | **Zanardo, V** [126] | **2016** | **Italy** | **Care** | **PC** | **uni** | **154 – 500** | **33.1 (4.7)** | **56** | **NR** | **MIBS** |
| 128 | Zdolska-Wawrzkiewicz,A [127] | 2018 | Poland | Care | CS | uni | 165 | 30.7 (4.3) | 77 | NR | MFAS |
| 129 | Zeitlin, D [128] | 1999 | UK | Care ^e^ | CS | uni | 38 | 26.5 | NR | 68 | BPNB |
| 130 | Zhang, H [129] | 2017 | China | Care | PC | uni | 255 | 29.8 | 100 | NR | MAI |
| 131 | Zimerman, A[130] | 2003 | Canada | Open | CS | uni | 171 – 231 | 31.6 | 63 | NR | MAAS |

All studies in **bold** were of fair methodological quality according the Quality Assessment Tool for Observational Cohort and Cross-sectional studies of the National Heart, Lung, and Blood Institute (other studies were of poor quality).

^a^ Setting is categorized in open population e.g. recruitment at websites, prenatal classes (Open), recruitment through primary care providers (Primary), recruitment through secondary care providers (Secondary), and in recruitment through primary, secondary and tertiary care providers (Care)

^b^ This study was partly supported by grants from the Pharmaceutical industry

^c^ The design of the analyses of factors of mother-to-infant bonding, is categorized in prospective (PC) and in cross-sectional (CS) design of the analyses. In several studies cross-sectional as well as prospective analyses were performed.

^d^ Nulliparae are women who expect their first child, or gave birth to their first child (when measured postnatally).

*^e^* Several analyzes have been reported in this article. Only the analyzes related to the general population were extracted.

Abbreviation: NR = not reported
