# Supplementary material for: Correlates of prenatal and postnatal mother-to-infant bonding quality: A systematic review
Source: PLoS One. 2019 Sep 24;14(9):e0222998. doi: 10.1371/journal.pone.0222998 (PMC6759162; doi:10.1371/journal.pone.0222998)
Supplement: S3 Table — (DOCX) [file pone.0222998.s007.docx]

**S3 Table** Overview of all identified studies, identified by number, examining associations between correlates examined in less than ten different studies with mother-to-infant bonding quality in the prenatal and postnatal period.

|  | **Prenatal mother-to-infant bonding** | | | **Postnatal mother-to-infant bonding** | | | |
| --- | --- | --- | --- | --- | --- | --- | --- |
| **Correlates** | **Association**  **YES** | **Association**  **NO** | **Inconclusive** | | **Association**  **YES** | **Association**  **NO** | **Inconclusive** |
| *Demographic* |  |  |  | |  |  |  |
| Ethnicity | 36, 80 | **39**, 69, 88, 116 |  | |  | 13, 71 |  |
| Nationality | 9 | 33 |  | |  | 91 |  |
| Socioeconomic status | **39** | 20, 21, 55, 124 |  | |  | 18, 91 |  |
| Joint family |  |  |  | |  | 2, **30**, 33, **41**, 87 |  |
| Living with partner | **39**, 67, 80, 88 | 24, | 69 | |  | 33, **59** |  |
| Length of partner relationship | 20, 24, 98, 124 | 67 |  | |  | 2 |  |
| Recruitment setting |  | 24 |  | |  |  |  |
| Amount of land by the household |  |  |  | |  | **30** |  |
| Household expenditure on food |  |  |  | |  | **30** |  |
| Insurance / social security |  |  |  | | 2 | 71 |  |
| Religion | 88 | **39**, 116 |  | |  |  |  |
| Job hours |  |  |  | |  |  | 113 |
| Economic dependence | 88 |  |  | |  |  |  |
| Parents alive |  | 3 |  | |  |  |  |
| Proportion of mothers’ income to family  income |  | 3 |  | |  |  |  |
| Mother born naturally |  | 3 |  | |  |  |  |
| Residence |  |  |  | |  | 91 |  |
| *Reproduction-related* |  |  |  | |  |  |  |
| Perinatal loss in history | 4 | 3, 40, 53, 74, **98,** 114 |  | | 12 | **8** |  |
| Health behavior | 69 | 89, 108 | 118 | |  | 87, 91 | **97** |
| Birth interval years |  |  |  | |  | 87 |  |
| Fetal count (singleton/twins) | 64 |  |  | |  |  |  |
| Expected fetal sex | 122 | 55, 80 |  | |  |  |  |
| Quickening | 67 | 3 |  | |  |  |  |
| Pregnancy health status |  | 2, **39**, 55, 69, 115 |  | |  |  | 75 |
| Pregnancy health | 42 | 16, 53 |  | |  | 2, 87, 113 |  |
| Oxytocin level in pregnancy |  |  | 68 | |  |  |  |
| Obstetrical problems in history |  | 20, 88 |  | |  | 33 |  |
| Infertility treatment | **17** | 3, 53, 66, 107 |  | | **17** | **51** |  |
| Hospitalization in pregnancy |  | 55 |  | |  | 2 |  |
| Maternal weight (gain)/ BMI |  | 108 | 44 | |  |  |  |
| Prenatal class attendance | 55 |  |  | |  | 2 |  |
| Ultrasound | 67 |  |  | |  |  |  |
| Known gender | 3 |  |  | |  |  |  |
| Intention to breastfeed | 34 | 43 | 65 | |  |  |  |
| Site midwifery practice | 80 | 71 |  | |  |  |  |
| Adequacy of prenatal care |  | 108 |  | |  |  |  |
| Societal expectations about pregnancy  role (IPRS) | 119 |  |  | |  |  |  |
| Self-perception in pregnant role (SPPRS) | 119 |  |  | |  |  |  |
| Being informed about postpartum health  care |  |  |  | |  |  | 2 |
| Sources of information during pregnancy |  |  |  | |  |  | 2 |
| Informed choice |  |  | **98** | |  |  |  |
| Informed participation in screening |  |  | **98** | |  |  |  |
| Down syndrome in history | 131 |  |  | |  |  |  |
| Regarding the fetus as a person | 131 |  |  | |  |  |  |
| *Birth-related* |  |  |  | |  |  |  |
| Pain at labour/ birth/ postpartum |  |  |  | |  | 33 |  |
| Perception of birth |  |  |  | | 6, **41** |  |  |
| Imminent preterm labour | 47 |  |  | |  | 47 |  |
| *Postpartum-related* |  |  |  | |  |  |  |
| Breastfeeding |  |  |  | | 1, 18, 5 | 2, 33, 87, 113, 120 |  |
|  | **Prenatal mother-to-infant bonding** | | | | **Postnatal mother-to-infant bonding** | | |
| **Correlates** | **Association**  **YES** | **Association**  **NO** | **Inconclusive** | | **Association**  **YES** | **Association**  **NO** | **Inconclusive** |
| Postpartum health hemorrhage en hb |  |  |  | | 2 | 87, 96 |  |
| Maternal sleep |  |  |  | |  |  | 113 |
| Touching (<60/>60) |  |  |  | |  | 13, 33 |  |
| Holding (<60/>60) |  |  |  | |  | 2, 13, 33 |  |
| *Psychosocial* |  |  |  | |  |  |  |
| Stress | 15, 21, 55, 88 |  | 116 | | **20,** 102 | **97,** 91 |  |
| Maternal mood blues (postpartum) |  |  |  | | 121 |  | **60, 111** |
| Physical/domestic violence |  | 92 |  | | **59** | **30,** 87, 129 |  |
| Adverse life events | 55 | 20, 56 | 101 | |  | 33 |  |
| Social desirability |  |  |  | |  |  | 12 |
| Psychological problems in history |  | 53 |  | | **29** | 33, 87 |  |
| Alexithymia |  |  | 24 | |  |  |  |
| Spirituality |  |  |  | |  | 6 |  |
| Mental well-being (combination anxiety  depression) | 20, 32, 117 |  | **98** | | **30** |  |  |
| Family functioning | 36 |  |  | |  |  |  |
| Mother and baby interaction |  |  |  | | 52 |  |  |
| Separation anxiety |  |  |  | |  | 12 |  |
| Women's attentional bias towards  distressed infants |  |  |  | | 90 |  |  |
| Support from partner during delivery |  |  |  | |  | 33 |  |
| Pregnancy related anxiety | 117 | 4 |  | | **28** |  |  |
| Childhood abuse |  |  |  | | **45,** 82, |  |  |
| Maternal sensitivity |  |  |  | | 105 |  |  |
| Maternal self-efficacy |  |  |  | | **41** |  |  |
| Self-esteem |  | 21 |  | |  | 91 |  |
| Self-concept |  | 37 |  | |  |  |  |
| Concern about physical appearance | 62 |  |  | |  |  |  |
| Maternal antenatal orientation |  |  |  | | 12 |  |  |
| Body image | 62 | 44 |  | |  |  |  |
| Quality of mother-in-law relationship |  |  |  | |  | **30** |  |
| Incarceration |  | 56 |  | |  |  |  |
| Premenstrual mood change |  | 53 |  | |  |  |  |
| Interpersonal reactivity index | 131 |  |  | |  |  |  |
| Empathic concern | 131 |  |  | |  |  |  |
| Caregiving to other adult | 117 |  |  | |  |  |  |
| Social phobia |  | 61 |  | |  | 61 |  |
| Sharing information on Facebook | 48 |  |  | |  |  |  |
| *Child-related* |  |  |  | |  |  |  |
| Birth weight |  |  |  | |  | **30**, 33, 87 |  |
| Congenital anomalies |  |  |  | |  | 10 |  |
| Infant temperament |  |  |  | | 102 |  | **29**, 83 |
| Child executive functioning |  |  |  | | **19** |  |  |
| Intensive care admission |  |  |  | | 33 |  |  |
| Neonatal problems |  |  |  | | 2, 33, 54 |  |  |
| Gestational age (at birth) |  |  |  | | 125 | **30**, 33, | **46** |
| Infant age |  |  |  | | 12 | **41**, 71, 90, 113 |  |
| Day care |  |  |  | |  | 113 |  |
| Colic |  |  |  | | 123 |  |  |
| Infants distinguishing between mothers  and others |  |  |  | | 2 |  |  |
| *Partner-related* |  |  |  | |  |  |  |
| Paternal fetal attachment | 100,**103**,115 |  | 93 | |  | 31 | **19** |
| Paternal general attachment |  |  | 20, 101 | |  | 12 |  |
| Couvade syndrome |  | 100 |  | |  |  |  |
| Partners employment |  | 115 |  | |  | 2, **59** |  |
| Partners age | 115 |  |  | |  | 2 |  |
| Partners education | 23, 115 |  |  | |  | 2 |  |
| Paternal desired pregnancy |  |  |  | |  | **60** |  |
| Number of children for partner | 115 |  |  | |  |  |  |
| Infants gender fathers preference |  | 23 |  | |  |  |  |
| Paternal stress |  |  |  | | **19** |  |  |

Studies in **bold** were of fair methodological quality according the Quality Assessment Tool for Observational Cohort and Cross-sectional studies of the National Heart, Lung, and Blood Institute (other studies were of poor quality).

The numbers refer to the different studies. The references are described in Appendix S3. More detailed information about the included studies are reported in Table S2.

The alpha level was set at 0·05 to be classified as an association

In some studies, multiple analysis were performed (e.g. at different time points, with different instruments)
